# Therapeutic targeting of the E3 ubiquitin ligase SKP2 in T-ALL

**DOI:** 10.1038/s41375-019-0653-z

**Published:** 2019-11-26

**Authors:** Sonia Rodriguez, Christina Abundis, Francesco Boccalatte, Purvi Mehrotra, Mark Y. Chiang, Mary A. Yui, Lin Wang, Huajia Zhang, Amy Zollman, Ricardo Bonfim-Silva, Andreas Kloetgen, Joycelynne Palmer, George Sandusky, Mark Wunderlich, Mark H. Kaplan, James C. Mulloy, Guido Marcucci, Iannis Aifantis, Angelo A. Cardoso, Nadia Carlesso

**Affiliations:** 1 Beckman Research Institute, Gehr Leukemia Center, City of Hope, Duarte, CA 91010 USA; 2 Herman B Wells Center, Indiana University Simon Cancer Center, Indiana University School of Medicine, Indianapolis, IN 46202 USA; 3 Department of Pathology and Perlmutter Cancer Center, NYU Langone Medical Center, New York, NY 10016 USA; 4 Department of Internal Medicine, University of Michigan School of Medicine, Ann Arbor, MI 48109 USA; 5 Division of Biology and Biological Engineering, California Institute of Technology, Pasadena, CA 91125 USA; 6 Department of Pathology, Indiana University School of Medicine, Indianapolis, IN 46202 USA; 7 Division of Experimental Hematology and Cancer Biology, Cincinnati Children’s Hospital Medical Center, Cincinnati, OH 45229 USA; 8 Present Address: Clinical Research Division, Fred Hutchinson Cancer Research Center, Seattle, WA 98109 USA; 9 Present Address: Department of Genetics, Ribeirão Preto Medical School, University of São Paulo, Riberão Preto, São Paulo 14049-900 Brazil

**Keywords:** Targeted therapies, Acute lymphocytic leukaemia

## Abstract

Timed degradation of the cyclin-dependent kinase inhibitor p27^Kip1^ by the E3 ubiquitin ligase F-box protein SKP2 is critical for T-cell progression into cell cycle, coordinating proliferation and differentiation processes. SKP2 expression is regulated by mitogenic stimuli and by Notch signaling, a key pathway in T-cell development and in T-cell acute lymphoblastic leukemia (T-ALL); however, it is not known whether SKP2 plays a role in the development of T-ALL. Here, we determined that SKP2 function is relevant for T-ALL leukemogenesis, whereas is dispensable for T-cell development. Targeted inhibition of SKP2 by genetic deletion or pharmacological blockade markedly inhibited proliferation of human T-ALL cells in vitro and antagonized disease in vivo in murine and xenograft leukemia models, with little effect on normal tissues. We also demonstrate a novel feed forward feedback loop by which Notch and IL-7 signaling cooperatively converge on SKP2 induction and cell cycle activation. These studies show that the Notch/SKP2/p27^Kip1^ pathway plays a unique role in T-ALL development and provide a proof-of-concept for the use of SKP2 as a new therapeutic target in T-cell acute lymphoblastic leukemia (T-ALL).

## Introduction

Acute lymphoblastic leukemia (ALL) is the most common pediatric cancer and second leading cause of childhood death [1]. Despite marked clinical advances in the treatment of the T-cell subtype of ALL (T-ALL), about 30% of patients experience relapse or refractory disease, which is associated with poor prognosis. Long-term survivors also suffer from debilitating complications. Thus, there is a need for innovative, curative therapeutic strategies for T-ALL, in particular relapse and refractory T-ALL [1].

Multiple oncogenic signaling mechanisms are involved in T-ALL, including hyperactivation of the PI3K-AKT pathway, deletion of key cell cycle inhibitors, ectopic expression of transcription factors, and hyperactivation of the Notch signaling pathway [2]. Although the identification of activating mutations in the majority of T-ALL patients places Notch signaling as a central player in T-cell leukemogenesis in both children and adults [3, 4], its therapeutic targeting poses significant challenges due to its important function in multiple organs [5]. Thus, it would be relevant to identify critical cell-context restricted downstream mediators of Notch, that could be targeted.

Previous studies in our laboratory demonstrated that Notch activation drives transcriptional activation of *Skp2*, promoting rapid cell cycle entry in myeloid cells [6]. SKP2 (S-phase kinase-associated protein 2) is the F-box protein of the E3 ubiquitin ligase complex and is responsible for targeted recognition and degradation of various cyclin-dependent kinases inhibitors (CKI), such as p21^Cip1^, p27^Kip1^, and p57^Kip2^ [7]. Thus, SKP2 is the major ubiquitin ligase that controls abundance of cell cycle regulatory proteins at the G_1_–S transition. SKP2 inactivation induces CKI accumulation and cell cycle arrest in cells and enhances hematopoietic stem cell (HSC) quiescence in vivo [8]. Conversely, SKP2 overexpression results in rapid cell cycle entry and is frequently associated with poor prognosis in cancers, including leukemia and lymphoma [9]. Indeed, the SKP2 target p27^Kip1^ is a critical regulator of T-cell proliferation; its accumulation is critical for restraining cell cycle at steady state, whereas its degradation is necessary for cell cycle entry in response to IL-7 and other cytokines [10–12]. Given the relevant role of p27^Kip1^ in T-cells and the converging effects of Notch and mitogenic stimuli on SKP2, we sought to determine the role of SKP2 in T-ALL leukemogenesis.

Here we show that genetic ablation of *Skp2* increases survival and significantly delays T-ALL progression in vivo, and that pharmacological blockade of SKP2 inhibits proliferation of human T-ALL cells. Taken together, our data support the rationale for the development of SKP2 inhibitors as therapeutic agents for T-ALL.

## Material and methods

### Mice

Twelve-week-old C57BL/6J *Skp2*^*−/−*^ mice backcrossed [8, 13]; Mx1Cre*Rbpj*^*lox/lox*^ mice [14]; 8-week-old B6.SJL-PtrcaPep3b/BoyJ (BoyJ; CD45.1), 20-week-old NOD/SCID and NSG (NOD *scid* gamma) mice were used as recipients for transplants (similar numbers of female/male were used). Mouse care and experimental procedures were performed in accordance with established institutional guidance and approved protocols of the Institutional Animal Care and Use Committees at Indiana University and City of Hope.

### Retroviral transduction, primary mouse leukemias, and xenograft models

Primary mouse leukemias were generated by retroviral transduction/transplantation approach [15]. Viral supernatant containing MSCV-GFP, MSCV-ICN/GFP, or MSCV-ΔEGFΔLNRΔP-GFP constructs [16] were used to transduce lineage negative (Lin^−^) progenitors from 12-week-old *Skp2*^*+/+*^, *Skp2*^*+/−*^, and *Skp2*^*−/−*^ CD45.2 mice. 2.5 × 10^4^ GFP^+^ cells/mouse admixed with 10^5^ protective BM cells from C57BL/6J (CD45.2) were transplanted into lethally irradiated (12 Gy) BoyJ CD45.1. Engraftment, GFP positivity, and T-cell content were evaluated at 2-week intervals in the PB. For secondary transplants, 0.5 × 10^6^ leukemic cells from primary transplants admixed with 10^5^ protective BM cells from C57BL/6J were transplanted into lethally irradiated BoyJ; CD45.1.

Xenograft models were generated by transplanting 3 × 10^6^ TAIL7-ICN/GFP cells into NSG mice. Mice were evaluated weekly for blast content and disease progression.

### SKP2 inhibitors

The SKP2 inhibitor C1 [17] and C25 [18] (MedChemExpress), were used to inhibit SKP2 at concentrations from 0–80 μM. IC50 dose (C1: 2.5 µM, C25: 30 µM) was used for cell cycle, apoptosis, and western blot analysis.

For xenografts models, C25 compound was synthesized by the Medicinal Pharmacy Core at COH. C25 was dissolved in sunflower oil and administered 3 days/week for 4 weeks by oral gavage (50 mg/kg).

### Bioinformatic analysis

Skp2 expression in mouse thymic and peripheral T-cell populations was performed with data from the Immunological Genome Project [19].

RNA-sequencing data for B-ALL, ETP-ALL, and T-ALL was taken from TARGET, GSE42328, and GSE57982.

Additional experimental methods and details are provided in Supplementary Materials, including a list of antibodies and primers used (Table S1 and S2, respectively).

## Results

### SKP2 is dispensable for T-lymphoid development in mice

**Fig. 1:**
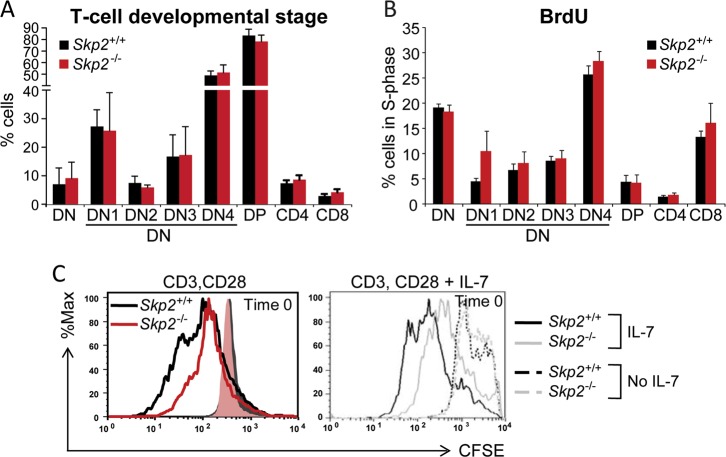
Impact of SKP2 depletion on normal lymphopoiesis. **a** Flow cytometry analysis of thymic subsets in *Skp2*^+/+^ and *Skp2*^*−/−*^ mice. Percentage of DN, DP, CD4-SP, and CD8-SP cells within the whole thymus and of DN1-4 within the DN subpopulation; *n* = 6 (*Skp2*^*−/−*^) and *n* = 9 (*Skp2*^+/+^) in three independent experiments. **b** Percentage of cells in S-phase measured by BrdU incorporation in indicated T-cell subsets in *Skp2*^+/+^ and *Skp2*^*−/−*^ thymocytes; *n* = 4 (*Skp2*^*−/−*^) and *n* = 7 (*Skp2*^+/+^) in two independent experiments. **c** Naive T-cells from *Skp2*^+/+^ and *Skp2*^*−/−*^ mice stained with CFSE (10 µM) were activated with anti-CD3 (2 µg/mL) and anti-CD28 (0.5 µg/mL) for 2 days (left panel). On day 3, an equal number of cells were CFSE stained and further treated for 72 h with 5 ng/ml of IL-7 (right panel). Black and red or gray lines show CFSE dilution in the *Skp2*^+/+^ and *Skp2*^*−/−*^ T-cells, respectively. Solid or dashed histograms show equal loading/Time 0 of the CFSE dye (representative experiment, *n* = 3). In bar graphs, results are shown as average ± SD

We have previously shown that Notch activation can directly regulate cell cycle entry by inducing p27^Kip1^ degradation via expression of the E3 ubiquitin ligase complex subunit SKP2 [6]. Given the critical role of p27^Kip1^ in timing cell cycle entry during T-cell development [20], we assessed the role of SKP2 in T-cell differentiation. Analysis of *Skp2* transcripts in different mouse organs revealed a significant expression of *Skp2* in bone marrow (BM) and thymus (Fig. S1A, left panel). In primary thymocytes, *Skp2* expression was dynamically regulated during thymocyte development, with higher levels of expression associated with high proliferative status, especially at post-β-selection double negative (DN; CD4^−^CD8^−^) stages, DN3B and DN4; and the immature CD8^+^ single positive (ISP) stage (Fig. S1A; right panel; reviewed in [21]). Given the premise that p27^Kip1^ downregulation is required for T-cell differentiation from DN to double positive (DP; CD4^+^CD8^+^) [22], and that loss of SKP2 results in p27^Kip1^ accumulation and cell cycle arrest [13], we anticipated that absence of SKP2 would compromise thymocyte differentiation in *Skp2* null mice. Surprisingly, despite efficient deletion of *Skp2* in the hematopoietic tissues ([13]; Fig. S1B), *Skp2*^*−/−*^ mice exhibited frequencies of DN and DP comparable with *Skp2*^*+/+*^ mice (Fig. 1a and S1D), showed normal sized thymus, and had similar numbers of total thymocytes (Fig. S1C). Thymocyte cell cycle activity was also comparable in *Skp2*^*−/−*^ and *Skp2*^*+/+*^ mice across various developmental stages (Fig. 1b). Mature lymphoid populations in the spleen of *Skp2*^*+/+*^ mice exhibited *Skp2* expression but no significant differences in total numbers of CD3^+^, γδ, and NK1.1 lymphocytes were found in absence of *Skp2*, although a trend for decrease was observed (Fig. S1E, F). Interestingly, our analysis of T-cell proliferative responses to common mitogenic signals showed that *Skp2*^*−/−*^ mature T-cells exhibited markedly impaired responses to CD3 plus CD28, and to IL-7 stimulation (Fig. 1c), highlighting the importance of SKP2 in the cell cycle entry induced by CD28 co-stimulation, as previously reported [23]. Taken together, these data show that SKP2 expression is dispensable for T-cell development while it may be required in mature cells to fully respond to mitogenic stimulation.

### SKP2 expression in thymocytes is upregulated by Notch signaling activation

**Fig. 2:**
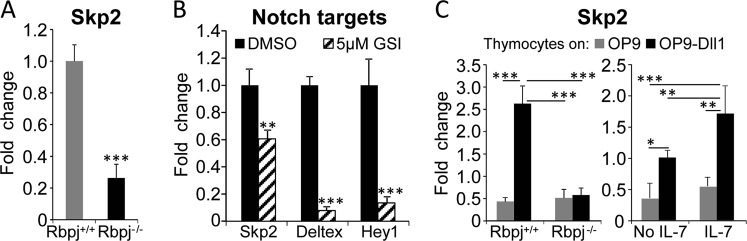
Activation of Notch and IL-7 signaling pathways cooperate to heighten SKP2 expression. **a** Fold change for *Skp2* mRNA expression measured by qRT-PCR in fresh population of total thymoctes harvested from *Rbpj*^*+/+*^ or *Rbpj*^*−/−*^ mice (Day 0). Results are indicated as average ± SE; *n* = 6 (*Rbpj*^*−/−*^) and *n* = 9 (*Rbpj*^*+/+*^) in two independent experiments. **b** Fold change for *Skp2*, *Deltex*, and *Hey1* mRNA expression measured by qRT-PCR in total thymocytes at 24 h of co-culture with OP9-Dll1 cells in the presence of DMSO or 5 µM GSI; each group *n* = 4. **c** Fold change for *Skp2* mRNA expression measured by qRT-PCR in two independent experiments. Left panel: *Rbpj*^*+/+*^ or *Rbpj*^*−/−*^ total thymocytes population at 4 days of co-cultured with OP9 or OP9-Dll1 cells in the presence of IL-7 (25 ng/ml); *n* = 3 (*Rbpj*^*−/−*^*)* and *n* = 4 (*Rbpj*^*+/+*^). Right panel: total thymocytes at 24 h of co-culture with OP9 or OP9-Dll1 cells in the absence or presence of IL-7 (25 ng/ml); *n* = 3 (OP9*)* and *n* = 4 (OP9-Dll1). All results are expressed as average ± SD. *p < 0.05, **p < 0.01, ***p < 0.005 by *t*-test or one-way ANOVA

Next, we defined the relationship between Notch and *Skp2* expression in thymocytes. *Skp2* expression was significantly reduced, although not completely abrogated, in thymocytes freshly isolated from mice lacking Notch downstream DNA-binding transcriptional factor RBPJ (*Rbpj*^*−/−*^; Recombination signal Binding Protein for immunoglobulin kappa j region; Fig. 2a). Similarly, expression of *Skp2* in *Rbpj*^*+/+*^ thymocytes was significantly decreased but not completely abrogated by pharmacological inhibition of Notch signaling by a γ-secretase inhibitor in vitro (GSI; Fig. 2b). Indeed, while GSI treatment almost completely inhibited expression of additional Notch targets *Hey1* and *Deltex* (86% and 92% inhibition, respectively), it inhibited only 40% expression of *Skp2*, suggesting that other pathways may contribute to *Skp2* expression at physiological state. Of note, a similar pattern of response for SKP2 and other Notch targets was also observed when GSI was used in vivo (Fig. S2A).

Although *Skp2* expression was moderately influenced by Notch inhibition, it was greatly affected by Notch activation. Following 4 days in culture, *Skp2* expression in *Rbpj*^*+/+*^ thymocytes dropped to the same levels observed in *Rbpj*^*−/−*^ thymocytes when in absence of Notch stimulation, but it was strongly upregulated when in presence of Delta1 (Dll1) ligand (sixfold increase; Fig. 2c; left panel). Accordingly, cell cycle analysis showed a sharp increase (up to fourfold) in percentage of *Rbpj*^*+/+*^ thymocytes progressing into S-phase when exposed to Dll1 ligand (Fig. S2B). Of note, some levels of *Skp2* expression and mitogenic response were preserved in *Rbpj*^*−/−*^ thymocytes in the absence of Notch signaling (Fig. 2c and S2B), suggesting Notch-independent *Skp2* regulation. As the culture conditions included IL-7, a cytokine required for T-cells survival and proliferation and involved in p27^Kip1^ regulation [24–26], we measured the distinct contribution of IL-7 and Notch activation to *Skp2* expression. *Skp2* expression and thymocyte mitogenic activity were sustained above basal level by IL-7 alone and were significantly upregulated in the presence of Notch signaling (Fig. 2c; right panel). Overall, these observations show that *Skp2* expression is robustly upregulated by activation of Notch signaling in combination with IL-7 stimulation. Ultimately, the cooperative effect of Notch and IL-7 may have a critical role in leading to uncontrolled proliferation and leukemia transformation.

### Oncogenic Notch upregulates SKP2 expression in T-ALL cells in vivo

**Fig. 3:**
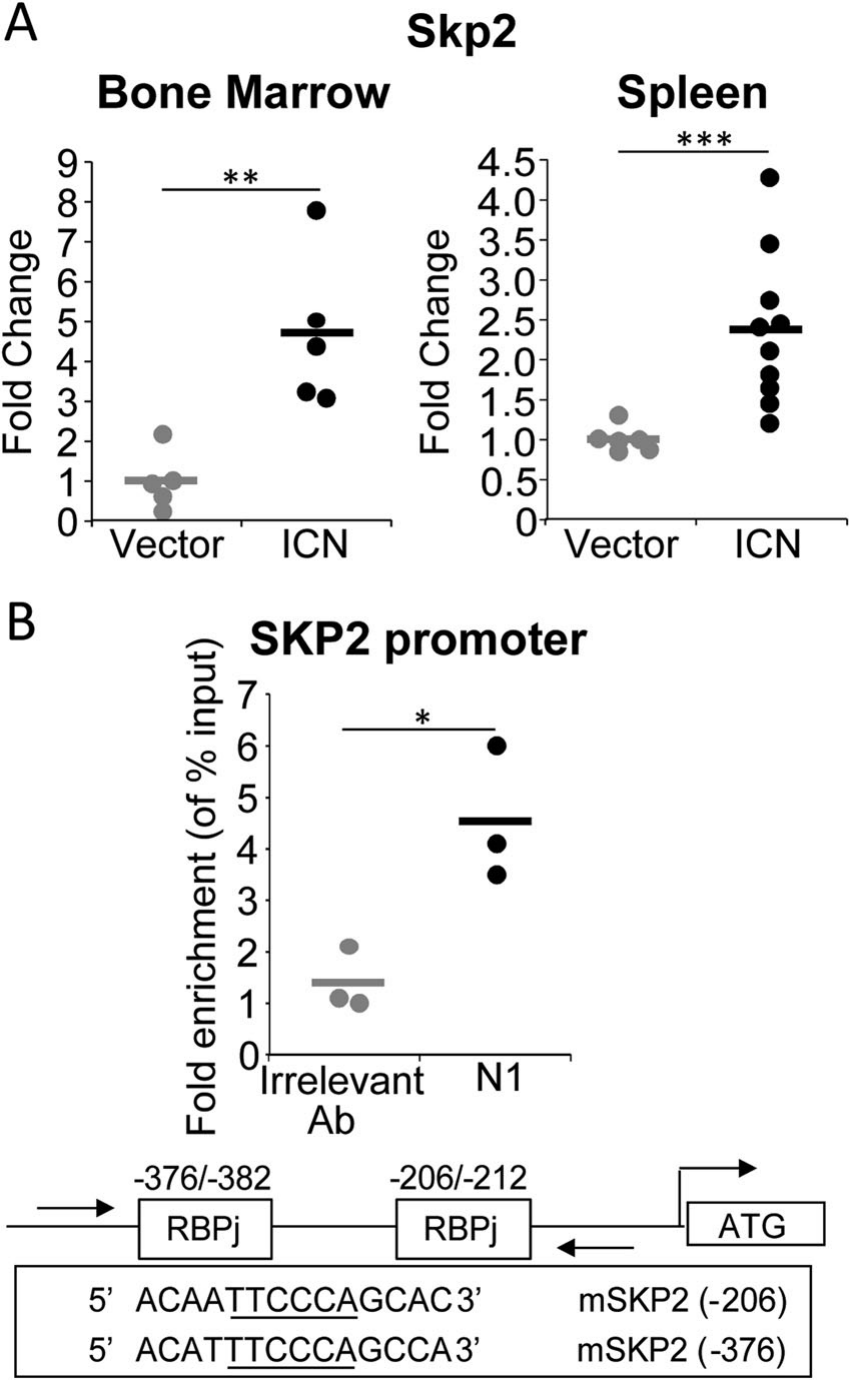
SKP2 expression is upregulated in vivo in the Notch-induced T-ALL model. Lin^−^ cells from C57Bl/6 mice (CD45.2) were transduced with MSCV-GFP (Vector) or MSCV-ICN1-GFP (ICN) constructs. 2.5 × 10^4^ ICN/GFP^+^ cells were transplanted into lethally irradiated BoyJ (CD45.1). Recipients were analyzed for: **a** Fold change for human *Skp2* mRNA expression measured by qRT-PCR in the BM (left panel; *n* = 5) and Sp (right panel; *n* = 6 (vector) and *n* = 10(ICN)). **b** CHIP assay. DNA isolated from the thymus of mice transplanted with ICN-GFP^+^ cells was immunoprecipitated with anti-Notch1 (N1) or anti-Jagged1 (irrelevant) antibodies Top panel: Scatter graph shows fold changes in SKP2 promoter enrichment measured by qRT-PCR (each group *n* = 3). Bottom panel: schematic representation of the Notch/RBPj binding sites within the mouse *Skp2* promoter. All results are from two independent experiments. In scatter plots average is shown by a horizontal line.*p < 0.05, **p < 0.01, ***p < 0.005 by *t*-test

Given the role of Notch in T-cell leukemogenesis and the converging effects of Notch and IL-7 on SKP2, we investigated the role of SKP2 in T-ALL leukemogenesis in vivo by using a Notch-induced T-cell leukemia mouse model. Overexpression of constitutively active intracellular form of Notch1 (ICN) in hematopoietic stem/progenitor cells (HSPCs) induces T-ALL with 100% penetrance [16, 27]. We transduced wild-type (WT; *Skp2*^*+/+*^) HSPCs with a retroviral construct containing ICN (MSCV-ICN/IRES/GFP; herein referred as ICN) or vector control (MSCV/IRES/GFP; herein referred as vector), and transplanted them into syngeneic WT mice. As anticipated, recipients showed accumulation of ICN-GFP^+^ and CD4^+^ CD8^+^ DP T-cells in BM (Fig. S3A, B) associated with rapid increase of white blood cell (WBC) counts, DP T-cells in peripheral blood (PB), and splenomegaly (Fig. S3C, D). HSPCs overexpressing ICN (Fig. S3E) showed a 5-fold and 2.5-fold increase in *Skp2* expression in BM and spleen, respectively, compared with HSPCs transduced with vector control (Fig. 3a). In these cells, *Skp2* upregulation was associated with direct binding of Notch1/RBPJ complex to the endogenous *Skp2* promoter (Fig. 3b and S3F).

### Loss of SKP2 antagonizes Notch-induced leukemia

**Fig. 4:**
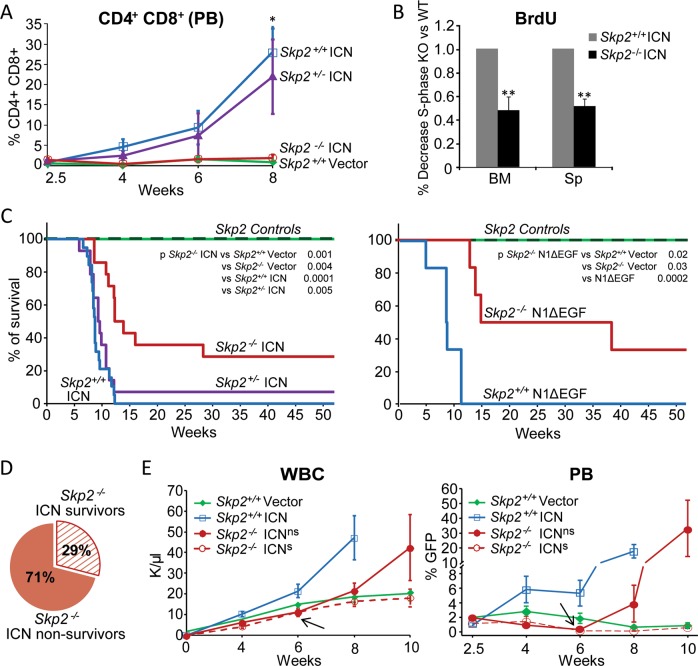
Loss of SKP2 attenuates Notch-induced leukemia. Lin^−^ cells from *Skp2*^+/+^, *Skp2*^+/−^, or *Skp2*^*−/−*^ (CD45.2) mice were transduced with MSCV-GFP (Vector), MSCV-ICN1-GFP (ICN), or MSCV-N1ΔEGFΔLNRΔP-GFP (N1ΔEGF) constructs. 2.5 × 10^4^ GFP^+^ cells were transplanted into lethally irradiated BoyJ (CD45.1) recipients (three independent experiments) and were analyzed for: **a** Percentage of DP T-cells in in gated CD45.2^+^ donor cells in PB. *Skp2*^+/+^ Vector (*n* = 6), *Skp2*^+/+^ ICN (*n* = 10), *Skp2*^+/−^ ICN (*n* = 10) and *Skp2*^*−/−*^ ICN (*n* = 6); (average ± SE). **b** Bar graph show BrdU incorporation in GFP^+^ cells from *Skp2*^*+/+*^ ICN (*n* = 5) and *Skp2*^*−/−*^ ICN (*n* = 3) in BM and spleen populations analyzed in recipients at week 8 from transplant. **c** Kaplan–Meier survival curve. Control cohorts include: *Skp2*^*+/+*^ Vector group (*n* = 10; green solid line) and *Skp2*^*−/−*^ nontransduced and *Skp2*^*−/−*^ Vector group (*n* = 8; dark green dashed line); all these groups behaved identically. Experimental cohorts: in left panel include *Skp2*^*+/+*^ ICN (*n* = 19; blue line), *Skp2*^*−/+*^ ICN (*n* = 14; purple line) and *Skp2*^*−/−*^ ICN (*n* = 14; red line); in right panel includes *Skp2*^*+/+*^ N1ΔEGF (*n* = 6; blue line) and for *Skp2*^*−/−*^ N1ΔEGF (*n* = 6; red line). **d** Scheme representing the survival percentages of *Skp2*^*−/−*^ ICN^ns^ (solid red) and *Skp2*^*−/−*^ ICN^s^ (dashed red). Mice within *Skp2*^*−/−*^ ICN genotype were distributed in two groups based on their survival, here called *Skp2*^*−/−*^ ICN^ns^ (no survivors; 71%) and *Skp2*^*−/−*^ ICN^s^ (survivors; 29%). Recipients of all genotypes (in three independent experiments), were analyzed at full-blown disease or at 1 year from transplant. **e** Line graph in left panel represents counts of WBC in the PB of recipient mice at the time points indicated. Line graph in right panel represents percentage of GFP^+^ cells gated on CD45.2^+^ cells in the PB of recipient mice at the time points indicated (average ± SE). *Skp2*^+/+^ Vector (*n* = 6), *Skp2*^+/+^ ICN (*n* = 10), *Skp2*^*−/−*^ ICN^ns^ (*n* = 6), and *Skp2*^*−/−*^ ICN^s^ (*n* = 3). Arrows indicates point of divergence between *Skp2*^*−/−*^ ICN^s^ and *Skp2*^*−/−*^ ICN^ns^. All results are expressed as average ± SD unless otherwise indicated. *p < 0.05, **p < 0.01, ***p < 0.005 by two-sample *t*-test, *t*-test or log-rank test

Next, we overexpressed ICN in HSPCs lacking SKP2 and transplanted them into syngeneic WT mice. We compared cohorts of mice transplanted with *Skp2*^*+/+*^*, Skp2*^*−/−*^, and *Skp2*^*−/+*^ HSPCs overexpressing ICN-GFP. Homing and engraftment of *Skp2*^*−/−*^ cells to BM is similar to *Skp2*^*+/+*^ cells [8]. As expected, animals transplanted with *Skp2*^*+/+*^ ICN cells developed full-blown T-ALL by week 8 post transplant, whereas mice that received *Skp2*^*−/−*^ ICN cells exhibited WBC counts similar to animals transplanted with vector control and low levels of donor T-cells in the PB (Fig. 4a and S4A). Absence of SKP2 in ICN cells was associated with increased levels of p27^Kip^ in most cases (Fig. S4B), and correlated with a 50% reduction in cell cycle activity (Fig. 4b), which occurred mostly at early G1-S phase transition (Fig. S4C).

Long-term follow-up of transplanted mice showed that all recipients of *Skp2*^*+/+*^ ICN cells were terminal by week 12, whereas T-ALL development was significantly delayed in recipients of *Skp2*^*−/−*^ ICN cells, with 50% survival at week 12 (Fig. 4c left panel). Notably, 29% of *Skp2*^*−/−*^ ICN recipients did not show signs of leukemia at week 30 and survived more than 1 year without evidence of disease. Interestingly, loss of one copy of *Skp2* did not confer any survival advantage to recipients of heterozygous *Skp2*^*+/−*^ ICN cells and these mice developed T-ALL with kinetics similar to recipients of *Skp2*^*+/+*^ ICN cells (Fig. 4a, c, S4A). As the ICN allele used above models the rare t(7:9) translocation and encodes a potent transcriptional transactivator [16], we tested the Notch1ΔEGFΔLNRΔP construct (defined here as N1ΔEGF), which better models the leukemia-associated NOTCH1 mutations disabling the negative regulatory region and PEST domain commonly seen in patients, and exhibits a weaker transcriptional activity than ICN [28]. We observed that *Skp2*^*−/−*^ ΔEGF recipient mice had a greater survival probability (50%, 95% CI: 10–90) than *Skp2*^*−/−*^ ICN recipients (29%, 95% CI: 5–52) at 32 weeks post transplant (Fig. 4c right panel and S4D).

### SKP2 deletion leads to early exhaustion of ICN-GFP populations

As shown in Fig. 4c, among all recipients of *Skp2*^*−/−*^ ICN cells, one group (71%) did not survive longer than 32 weeks from transplant, whereas one group (29%) remained healthy and survived at long-term (scheme in Fig. 4d). To exclude the possibility that some *Skp2*^*−/−*^ ICN recipients did not survive because of low BM engraftment, we measured the levels of CD45.2^+^ donor cells in the PB at different times post transplant. As shown in Fig. S5A, the engraftment of CD45.2 donor cells was robust and similar in all *Skp2*^*+/+*^ and *Skp2*^*−/−*^ groups. To exclude the possibility of insufficient ICN expression by *SKP2*^*−/−*^ transduced cells in the *Skp2*^*−/−*^ ICN recipients that survived, we measured ICN expression in BM cells after transduction and soon after transplant. Prior to transplant, transduced *Skp2*^*−/−*^ HSPC-ICN, validated for *Skp2* deletion (Fig. S5B), showed expression of ICN comparable with transduced *Skp2*^*+/+*^ and *Skp2*^*+/−*^ HSPCs (Fig. S5C). In addition, BM sampling performed in an independent cohort showed that a similar number of *Skp2*^*+/+*^*, Skp2*^*−/−*^, and *Skp2*^*−/+*^ ICN-GFP^+^ transduced cells (~9 × 10^3^) engrafted in recipients at 2.5 weeks from transplant (Fig. S5D). To examine more closely the characteristics of the recipients of *Skp2*^*−/−*^ ICN cells, we separated and analyzed independently the group that survived and the group that did not survive (Fig. 4d), herein indicated as *Skp2*^*−/−*^ICN^s^ and *Skp2*^*−/−*^ICN^ns^, respectively. WBC counts and frequency of ICN-GFP^+^ cells were similar in the PB of *Skp2*^*−/−*^ ICN^ns^ and *Skp2*^*−/−*^ ICN^s^ recipients and followed the kinetics of *Skp2*^*+/+*^ vector control mice until week 6 of transplant (Fig. 4e). At this point, despite comparable initial levels of ICN expression (Fig. S5C, D) and low percentages of ICN-GFP^+^ in the PB (average 0.3% vs. 0.2%), *Skp2*^*−/−*^ ICN^ns^ recipients exhibited increasing levels of WBCs and of T-cell leukemia blasts, whereas *Skp2*^*−/−*^ ICN^s^ recipients showed WBC counts and T-cell percentages comparable with the control group (Fig. 4e). ICN transcripts were no longer detected in BM of *Skp2*^*−/−*^ ICN^s^ recipients at 1 year from transplant, whereas elevated ICN transcripts were detected in BM of leukemic *Skp2*^*−/−*^ ICN^ns^ recipients at time of sacrifice when moribund (Fig. S5E). Notably, *Skp2*^*−/−*^ ICN^s^ recipients exhibited normal hematopoiesis and did not show signs of leukemia at 1 year from transplant. This outcome was validated by the presence of normal myeloid and B-cells, normal frequency of T-cells and LSK cells in the BM (Fig. S6A), absence of splenomegaly and of leukemic infiltrates in spleen, liver, and bone, and tissue organization similar to matched control mice (Fig. S6B, C). In contrast, all *Skp2*^*−/−*^ ICN^ns^ recipients developed leukemia and showed PB, BM, and tissue involvement similar to that observed in recipients of *Skp2*^*+/+*^ ICN cells; however, they exhibited a delayed onset of disease and slower kinetics of progression (Fig. 4c–e), which persisted in secondary transplants (Fig. S6D).

Overall, these data demonstrate that absence of SKP2 can prevent leukemia development by driving exhaustion of *Skp2*^*−/−*^ ICN population in some recipients.

### Gene expression profiling reveals a Notch/IL-7/SKP2 signature in T-ALL samples

**Fig. 5:**
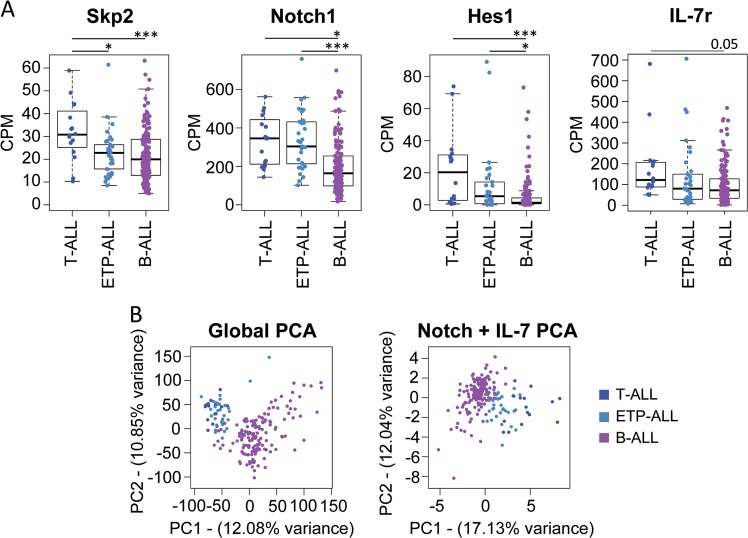
A Notch/SKP2/IL-7 signature defines human T-ALL samples. **a** Expression of SKP2, NOTCH1, HES1, and IL-7r transcripts (CPM) across primary T-ALL, ETP-ALL, and B-ALL cases (*n* = 17, 34, and 154, respectively). **b** Principal component analysis showing the distribution of primary T-ALL, B-ALL, and AML samples according to their whole transcriptome (left panel) or to the expression of 23 key genes involved in NOTCH and IL-7 signaling pathways (right panel)

SKP2 upregulation results in rapid cell cycle entry and is frequently associated with poor prognosis in cancers [18, 29], including leukemia and lymphomas [9, 30]. We performed a bioinformatic analysis of publicly available transcriptome data and found that patients with T-ALL exhibited higher expression of *Skp2* in comparison with B-ALL patients (Fig. 5a, far left panel). Not surprisingly, given the high frequency of Notch activating mutations [3, 26] and the role of IL-7 in T-ALL, this profile was associated with increased Notch activation and increased availability to respond to IL-7, as indicated by higher average expression of *Notch1* and its targets *Hes1* and *IL-7r*, respectively (Fig. 5a, left, right, and far right panels). In light of these data, we performed principal component analysis using *Skp2* expression in combination with other Notch and IL-7 signaling downstream targets (23 genes; Fig. S7A). We found that this combination was sufficient to separate the identity of T-ALL from B-ALL samples, as seen when using the global transcriptome (Fig. 5b). Collectively, these findings suggest a prominent role of the Notch/IL-7/SKP2 axis in T-ALL biology.

### Pharmacological blockade of SKP2 inhibits human T-ALL in vitro and in vivo

**Fig. 6:**
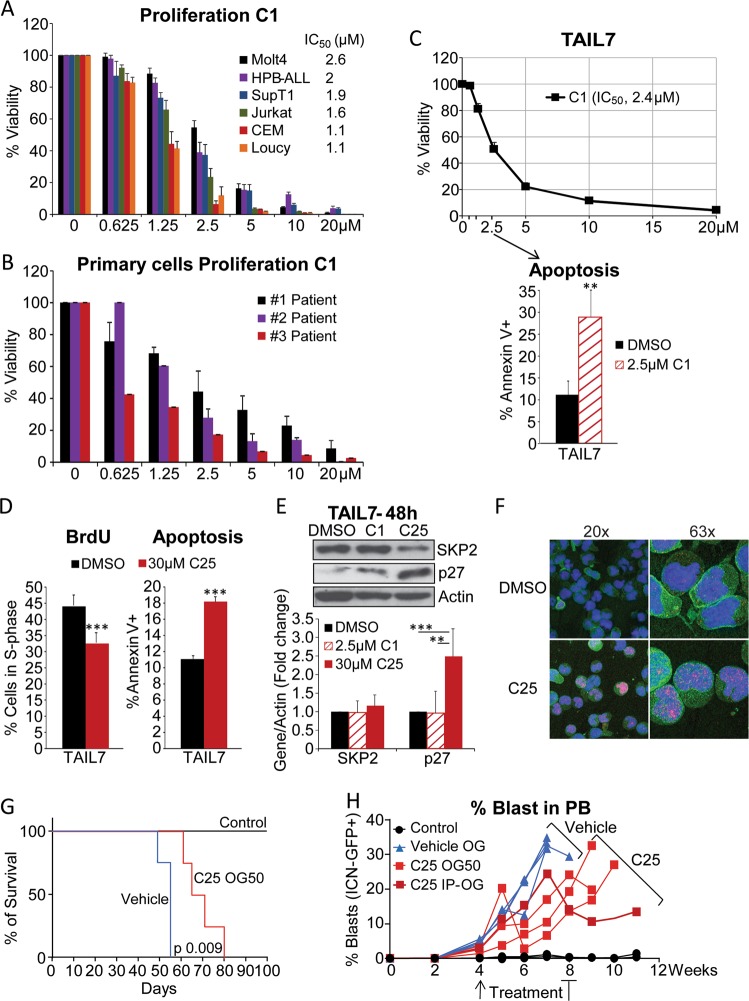
SKP2 blockade by small molecule inhibitors reduces proliferation of T-ALL in cell lines, primary cells, and in vivo in a xenograft model of T-ALL. Viability assays for the C1 inhibitor were performed at 96 h for cell lines and 48 h for primary cells. DMSO was used as vehicle control in all experiments. **a** Percentage viability of human T-ALL cell lines treated with increasing doses of the SKP2 inhibitor C1 (average ± SE). SupT1, HBP-ALL (*n* = 4), and all other cell lines (*n* = 6) in three independent experiments. **b** Percentage viability of primary T-ALL patient cells treated with increasing doses of the SKP2 inhibitor C1 (three patients samples, two independent experiment). **c** Top panel: percentage viability of TAIL7 cells treated with increasing doses of the SKP2 inhibitor C1 (*n* = 6 in three independent experiments). Bottom panel: percentage of cells in S-phase measured by BrdU incorporation and percentage of apoptotic cells measured by Annexin V in TAIL7 cells treated for 48 h with 2.5 µM C1 or DMSO; *n* = 4. **d** Percentage of cells in S-phase measured by BrdU incorporation and percentage of apoptotic cells measured by Annexin V in TAIL7 cells treated for 48 h with 30 µM C25 or DMSO as vehicle control (average ± SD); *n* = 3. **e** Western blot (top) and densitometry analysis (bottom) of SKP2, p27^Kip1^, and β-Actin protein levels in TAIL7 cells treated with DMSO, 2.5 µM C1 and 30 µM C25 for 48 h; four independent experiments. **f** Immunofluorescence images show accumulation and localization of p27^kip1^ (red) in the nucleus (blue) in TAIL7 cells following treatment with 30 µM of C25 or DMSO for 48 h (representative of three independent experiments). **g** Line graph shows average percentage of ICN-GFP^+^ blasts in the PB of each recipient mouse at the time points indicated. End point is the last point indicated in the graph. **h** Kaplan–Meier survival curve. All experiments are shown as average ± SD unless otherwise indicated. **p < 0.01, ***p < 0.005 by *t*-test. *p* = 0.009 by long rank test

Next, we investigated the effects of SKP2 pharmacological blockade on human T-ALL cells by using the small molecule SKP2 inhibitor, C1, developed for its ability to block protein–protein interaction between SKP2 and p27^Kip1^ [17]. First, we validated C1 compound’s selectivity by showing that C1 inhibited cell growth of *Skp2*^*+/+*^ without affecting significantly *Skp2*^*−/−*^ murine embryonic fibroblasts (Fig. S8A). C1 was very effective in inhibiting murine primary *Skp2*^*+/+*^ ICN T-ALL cells (IC_50_: 0.62 µM) while showing little toxicity on primary murine BM cells (Fig. S8B–D). Treatment of several human T-ALL cell lines, all exhibiting elevated SKP2 levels (Fig. S8E), showed a significant dose-dependent inhibition of cell proliferation in cell lines with high Notch activation and also in cell lines established from high-risk and relapsed patients, which have little or no Notch signaling, such as CEM and Loucy (Fig. 6a), suggesting that SKP2 inhibition is effective also in Notch-independent highly proliferative leukemia subtypes.

As IL-7 contributed to *Skp2* expression and cooperated with Notch activation in inducing cell cycle entry in healthy murine thymocytes (Fig. 2c; right panel), we validated these results in the IL-7-dependent T-ALL cell line, TAIL7 [31], and in primary T-ALL cells, which are responsive to IL-7 for survival and proliferation. A time-dependent increase of SKP2 levels, associated with significant reduction in p27^Kip1^ levels, was observed in TAIL7 cells following stimulation with IL-7 (Fig. S8F), highlighting the conservation of the IL-7/SKP2/p27^Kip1^ axis from mouse to human T-cells. We observed that C1 treatment significantly inhibited cell viability of primary T-ALL cells (Fig. 6b; IC_50_: 1.72 µM) and of TAIL7 cells (Fig. 6c; IC_50_: 2.4 µM) even in the presence of high-dose IL-7. This effect was also associated with a significant increase in the percentage of apoptotic cells (Fig. 6c). The effects of SKP2 pharmacological blockade were corroborated by using an additional SKP2 inhibitor, the C25 compound [18]. C25 inhibited TAIL7 viability in a dose-dependent manner (Fig. S9A) and it was effective in inducing cell cycle arrest and apoptosis (Fig. 6d) as well as leading to accumulation and localization of p27^Kip1^ in the nucleus (Fig. 6e, f).

Currently available SKP2 inhibitors have limited pharmacological properties and their use in vivo poses numerous challenges. To provide a proof-of-concept for the antileukemic effect of SKP2 inhibitors, we treated leukemic mice with the C25 compound, which was previously tested in vivo in a solid tumor model and showed some efficacy [18]. We transplanted TAIL7-ICN cells in NSG mice, as a more aggressive and traceable model, and delivered C25 treatment as monotherapy for 4 weeks, starting at evidence of circulating blasts (Fig. S9B). While the leukemic mice treated with vehicle succumbed to the disease at week 7, mice treated with C25 exhibited prolonged survival (Fig. 6g) and showed a significant decrease in blasts in PB and spleen (Fig. 6h and S9C–E). Collectively, these results show that SKP2 blockade impairs the growth of human T-ALL cells in vitro and in vivo and argue for development of SKP2 inhibitors with better pharmacological properties.

## Discussion

SKP2 (S-phase kinase-associated protein 2) is the F-box protein of the SCF E3 ubiquitin ligase complex that targets proteasome-dependent degradation of many CKIs [32–35], in particular phosphorylated p27^Kip1^, promoting rapid cell cycle entry in many cell types. In this study, we addressed the role of SKP2 in normal and malignant T-cells and showed that SKP2 is dispensable for normal thymocyte development but plays an active role in T-cell leukemogenesis.

We previously discovered that SKP2 is a direct target of Notch [6], which is required for T-cell development and is involved in T-cell leukemogenesis [36, 37]. The transcriptional program activated by Notch signaling is necessary for T-cell differentiation, but requires accurate regulation of cell proliferation and timed downregulation of p27^Kip1^ [20, 22]. We postulated that SKP2, being a key cell cycle regulator at the intersection of Notch and p27^Kip1^, would play a critical role in T-cell differentiation. Surprisingly, we found that although Notch signaling contributes significantly to T-lymphocytes cell cycle entry by inducing *Skp2*, *Skp2* expression appears to be dispensable for T-cell development. The dispensable role of SKP2 in T-cell development is in striking contrast with the critical role of another positive cell cycle regulator, cyclin D3, which loss severely impairs lymphoid development [38]. It is possible that while deletion of cyclin D3 completely disables CDK4 and CDK6 complexes, effectively impeding cell cycle progression, loss of SKP2 in thymocytes does not result in the sufficiently elevated levels of p27^Kip1^ required to block all cyclin/CDK complexes (CDK2, 4 and 6). Our results are in agreement with reports showing that coupling of proliferation and differentiation in T-cells is compromised only in transgenic mice overexpressing high, but not intermediate, levels of p27^Kip1^ [22], and that loss of p27^Kip1^ only partially rescues the cell cycle block induced by cyclin D3 deletion [39]. Taken together, these observations indicate that developing thymocytes may possess regulatory mechanisms that allow cell cycle progression notwithstanding increased levels of p27^Kip1^. Our data also suggest that these compensatory mechanisms may not be active in mature T-cells stimulated by mitogenic factors, as shown by impairment of cell proliferation in mature *Skp2*^*−/−*^ T-cells following stimulation with CD28 and IL-7. The mechanisms that influence these two distinct outcomes are unclear and warrant further investigation.

In this study, Notch inhibition by genetic deletion or pharmacological blockade showed that Notch signaling contributes only partially to maintenance of basal *Skp2* levels, while activation of Notch by high density Dll1 ligand or constitutive active Notch (ICN) upregulates substantially *Skp2* expression. This observation raises the possibility that supraphysiological stimulation of Notch1 signaling is required to license new oncogenic drivers that would not be sufficient or accessible with lower, physiological levels of Notch signaling. This is clinically relevant as clinical trials have shown that Notch inhibitors are too toxic due to Notch requirement in essential physiological functions. These observations raise the possibility of specifically targeting Notch in cancer by selectively disabling its critical downstream target genes, such as SKP2.

To determine the impact of SKP2 on leukemogenesis, we have used a Notch-induced murine leukemia model driven by the ICN allele, which models the rare t(7:9) translocation and is a potent transcriptional transactivator. It is noteworthy that in this model of aggressive T-ALL, SKP2 deletion prevented leukemia development in ~30% of mice, which were disease-free for the entire period of the follow-up (>1 year), and delayed onset of leukemia in two thirds of the transplanted cohort. Survival was further prolonged when *Skp2* was deleted in the context of a weaker form of activated Notch. Genetic deletion of *Skp2* in presence of ICN resulted in p27^Kip1^ accumulation and reduction of cell cycle activity in lymphoblasts, and correlated with various degrees of disease inhibition. The robust antileukemic effect of SKP2 deletion on some mice raises the possibility that SKP2 exerts tumor suppressive effects also through additional mechanisms other than cell cycle. Indeed, a role of SKP2 in regulating cell metabolism has been recently highlighted in breast cancer [40] and warrants future studies during leukemogenesis.

Interestingly, our experimental design revealed that despite equivalent deletion of SKP2 and similar expression of ICN, *Skp2*^*−/−*^ ICN cells were extinguished in some recipients while persisting and leading to leukemogenesis in others. This outcome did not seem to depend on the levels of ICN or SKP2, and it is likely influenced by different factors, including microenvironmental cues, immune responses, and other stochastic events. Previous studies show that supraphysiological levels of Notch signals gradually deplete HSPCs over weeks [41]. Thus, Notch-activated HSPCs seem to have only a limited window of time to initiate T-ALL. It is possible that during this window, in the context of SKP2 absence, some incipient leukemia cells were able to access SKP2-independent mechanisms to fully transform into T-ALL. Future studies are necessary to clarify the mechanisms that can override the impact of the cell cycle inhibitory effects of SKP2 deletion in T-ALL blasts and to determine the contribution of non-cell autonomous factors, such as BM microenvironment and the immune response. Given the potential role of SKP2 in cancer stem cells [18], elucidating SKP2-dependent versus SKP2- and p27^Kip1^- independent mechanisms will provide insights into the mechanisms of cancer cells resistance and relapse.

Our data show that IL-7 signaling synergizes with Notch activation in inducing *Skp2* expression and cell cycle activation in thymocytes and human T-ALL. Accordingly, Il-7Rα is an important downstream target gene of Notch [42–44]. It was previously reported that IL-7 stimulation induces p27^Kip1^ degradation and cell cycle entry in human T-ALL [10], and that SKP2 can mediate p27^Kip1^ degradation upon IL-7 stimulation in murine thymocytes [25]. Our observations uncover a previously unrecognized role for SKP2 as a point of convergence between Notch and IL-7 pathways in T-ALL. Not surprisingly, *Skp2* expression has been found elevated in lymphoid malignancies and in T-ALL cell lines [9, 11]. This feature may specifically reflect the biology of T-ALL blasts, in which high frequency of activating Notch mutations and dependence on IL-7 signaling cooperate in regulating *Skp2* expression and cell cycle activation, generating a T-ALL specific molecular signature. Indeed, a combination of a limited number of genes [37], which includes *Skp2* plus other Notch and IL-7 target genes, is sufficient to separate the identity of T-ALL from B-ALL, which also express *Skp2*, albeit at lower levels.

In our study, SKP2 inhibitors were effective in reducing viability of human T-ALL cell lines and primary T-ALL samples in vitro even in the presence of the protective effect of IL-7. Furthermore, despite the pharmacological limitations of C25 SKP2 inhibitor used in this study, significant reduction of tumor burden was observed with a short monotherapy regiment in an aggressive xenograft model of T-ALL.

Although current frontline therapies have demonstrated efficacy in T-ALL, 25–30% of patients remain refractory to therapy or undergo disease relapse. Furthermore, current therapies in pediatric patients have long-term toxicities. Thus, the identification of new and less toxic therapeutic approaches is an unmet clinical need. SKP2 inhibitors with better pharmacological profile have the potential to be highly effective in T-ALL, and likely in other cancers, as SKP2 expression has been shown to correlate with progression in different tumors and blood malignancies (review in [45]). Other possible advantages of SKP2 as a therapeutic target in cancer include the potential for selective targeting compared with the use of other signaling inhibitors with broader activities or higher toxicity, such as the proteasomal inhibitor bortezomib [46] or the CDK4/6 inhibitors [47]. One can predict that selective SKP2 inhibitors would have low toxicity since *Skp2*-knockout mice are healthy. Therefore, increasing efforts are necessary to develop more effective SKP2 inhibitors that can be used in preclinical studies and clinical trials. Our present study shows that T-ALL cells rely on the SKP2/p27^Kip1^ axis more heavily than normal T-cells for cell growth, and that SKP2 inhibition antagonizes T-cell leukemogenesis while resulting in little toxicity to normal cells, thus providing a proof-of-concept for targeting SKP2 in T-ALL leukemia.

## Supplementary information

Supplemental materials
