# Supplementary material for: Therapeutic targeting of the E3 ubiquitin ligase SKP2 in T-ALL
Source: Leukemia. 2019 Nov 26;34(5):1241–52. doi: 10.1038/s41375-019-0653-z (PMC7192844; doi:10.1038/s41375-019-0653-z)
Supplement: Supplementary file 1 — Supplemental materials [file 41375_2019_653_MOESM1_ESM.docx]

SUPPLEMENTARY MATERIALS:

**Cells and cell culture**

BM cells were harvested from femurs. Lineage positive cells were depleted (using Miltenyi Biotec depletion kit which includes the following antibodies: CD5, B220, CD11b, Gr1, 7/4 and Ter-119); Lineage negative cells (Lin^-^) were seeded at 2.5x10^5^ cells/ml in IMDM media supplemented with 10% FBS, SCF (50ng/ml), IL-3 (50ng/ml) and Flt3 (50ng/ml) in 24-well bio-coat plates and cultured at 37°C, 5% CO_2_ for retroviral transduction. Primary murine BM leukemic cells were cultured in the same conditions in the presence of IL-7 (10ng/ml).

Primary thymocytes were obtained from 10-15 week old mice and cultured in RPMI media supplemented with 10% FBS, SCF (50ng/ml), Flt3 (50ng/ml), and IL-7 (25ng/mL). When co-cultured with stroma cells, 2x10^6^ thymocytes were plated in 12-well plates together with 8x10^4^ stroma cells in α-MEM supplemented with 10% FBS and IL-7 (25ng/mL). In GSI experiments, 5µM GSI (GSI XXI, EMD-Calbiochem) was used.

OP9 and OP9-Delta1 cell lines were maintained in α-MEM supplemented with 20% FBS. MEFs were generated from E13-E15 embryonic day of each genotype (*Skp2^+/+^* and *Skp2^-/-^*) using standard methods([1](#_ENREF_1)) and were grown in DMEM media supplemented with 10% FBS and L-Glutamine (20μM).

All cell lines (except TAIL7) were purchased from ATCC. TAIL7 was directly obtained from Dr. Cardoso ([2](#_ENREF_2)) and checked periodically for IL-7 dependence. All cell lines were kept *in vitro* for not longer than 6 weeks, were periodically checked for the presence of the original phenotypic markers and were tested for mycoplasma contamination.

All cell lines (JKB198, TAIL7, SupT1, Jurkat, MOLT4, HBP-ALL, CEM, and Loucy) were maintained in RPMI1640 media supplemented with 10% FBS; 5ng/mL IL-7 (R&D) was added for TAIL7 cells. For IL-7 starvation and stimulation experiments, TAIL7 cells were maintained in medium with 2% FBS without IL-7 for 5 days and then stimulated with IL-7 (10ng/ml).

Primary patient samples were obtained from Dr. Mulloy (Cincinnati Children Hospital IRB approval #2010-0658). Primary patient samples were expanded in NOD/SCID mice, isolated from BM, and then cultured in RPMI supplemented with 10% FBS, SCF (10ng/ml), Flt3 (10ng/ml), IL-7 (10ng/ml), IL-9 (10ng/ml), and IL-15 (20ng/ml). All cytokines were purchased from Miltenyi Biotech or Peprotech.

The colony forming cell (CFU) assay was performed using 5000 BM cells/well in complete methylcellulose media from Stem Cell Technology. Colonies were read after 7 days.

**Flow cytometry analysis**

PB obtained from tail veins was labelled with anti-CD45.2, CD4, and CD8 antibodies to detect mouse cells, and with anti-hCD45 antibody to detect human cells. Mouse BM cells and thymocytes were labelled to identify developmental subpopulations as described in ([3](#_ENREF_3)); the complete list of antibodies used is in the Supplemental Material (Table S1). Cells were collected using LSRII or Accuri C6 flow cytometers (BD Biosciences; San Jose, CA) and analyzed using FlowJo.

**Cell cycle, proliferation, viability, and apoptosis analysis**

For cell cycle analysis thymocytes, BM and TAIL-7 cells were pulsed with Bromodeoxyuridine (BrdU; 10μM) for 3h prior to analysis. All cells were fixed and stained following the manufacturer’s instructions (BrdU-APC flow kit; BD Bioscience) in combination with surface markers.

Proliferation was determined by divisional history by CFSE (CarboxyFluorescein Succinimidyl Ester). Naïve CD4^+^ T-cells were isolated from spleen using magnetic selection (Miltenyi Biotec) and further activated with anti-CD3 (2µg/mL) and CD28 (0.5µg/mL). Cells were labeled with 10µM CFSE (Life technologies) as recommended by the manufacturer and analyzed by flow cytometry.

For viability assays, cells were seeded in 96-well plates at 2.5x10^4^ cells/well (cell lines) or at 2x10^5^ cells/well (primary cells) and analyzed using the Cell Titer-Glo luminescent cell viability assay (Promega) following the manufacturer’s instructions. Samples were measured in an Lmax luminometer (Molecular Devices; San Jose, CA).

Apoptosis was measured using APC-Annexin V and Propidium iodide (PI) or 7-AAD staining (BD Bioscience).

**Immunoblot analysis**

Protein extracts were prepared using RIPA buffer (50mM Tris pH 8, 150mM NaCl, 0.1% SDS, 1% NP40, 0.5% NaDeoxycholate) supplemented with protease inhibitor cocktail (Roche). Antibodies against p27, SKP2, Notch1 and β-Actin were purchased from Santa Cruz and Cell Signaling. β-Actin was used to normalize protein loading and Kodak easy share software or Image J were used for quantification (List of antibodies in Table S1).

**Quantitative RT-PCR analysis and chromatin immunoprecipitation (ChIP) assay**

Total RNA was isolated using Trizol (Invitrogen). cDNAs were generated by reverse transcription (SuperScript II kit; Bio Rad). RT-PCR reactions were performed as previously described([4](#_ENREF_4)) using GAPDH to normalize mRNA levels (List of primers in Table S2). Primers against specific sequences of human Notch1 were used to distinguish oncogenic ICN from endogenous murine *Notch1* expression.

ChIP assay was performed as previously described([5](#_ENREF_5)). Briefly, thymocytes from leukemic animals were fixed with 1% formaldehyde and quenched by adding 0.125M glycine. After sonication, samples were pre-cleared with protein A beads and immunoprecipitated with anti-Notch1 or anti-Jagged1 used as negative control (here called irrelevant), both purchased from Santa Cruz (List of antibodies in Table S1). After reversion of cross-link, DNA was extracted by phenol-chloroform and ethanol and analyzed by RT-QPCR on the SKP2 promoter region containing RBPj binding sites (List of primers in Table S2).

**Immunohistochemistry**

TAIL7 cells (8x10^4^) were placed in slides using cytospin (600 rpm 8’; Cytospin4 Thermo Scientific). After fixation with methanol, cells were stained with anti-p27, anti-CD45 and DAPI (Sigma Aldrich). Slides were imaged using (Zeiss LSM 880 with Airyscan; Ontario, CA) and analyzed with Zen 2.3 Lite (List of antibodies in Table S1).

**Bioinformatic analysis**

Analysis of Skp2 gene expression in mouse thymic or peripheral T-cells populations was performed using publicly available data downloaded from the Immunological Genome Project (www.immgen.org) using sorted cell subsets probed with Affymetrix Mouse Gene 1.0ST arrays([6](#_ENREF_6)).

RNA sequencing pre-analyzed data for B-ALL was downloaded as raw counts from the public available data TARGET Data Matrix “ALL Phase I” and “ALL Phase II” (https://ocg.cancer.gov/programs/target/data-matrix), which was analyzed with a similar pipeline than the T-ALL and ETP-ALL data.

RNA sequencing data for T-ALL and ETP-ALL was taken from two merged datasets: GSE42328 and GSE57982, (the latter containing only 10 T-ALL samples). The call for ETP-ALL and canonical T-ALL in GSE42328 and GSE57982, was based on a large clinical study([7](#_ENREF_7)). Briefly, based on the gene expression and immunophenotype data of 239 diagnostic T-ALL patients([7](#_ENREF_7)), we generated a matrix of 49 ETP-ALL specific genes and 73 T-ALL genes (from the top 140 differentially express genes), that we applied to the above dataset to segregate ETP and non-ETP cases.

The data was analyzed as outlined in the following: all sequencing reads were aligned against the human reference genome hg19 using the STAR aligner v2.5.0c([8](#_ENREF_8)). Only uniquely aligned reads were kept for further analyses. Read-counts overlapping annotations from Ensembl Genes V75 were generated using the STAR function “--quantMode GeneCounts”. Gene annotations that were missing in either the T-ALL or B-ALL dataset were discarded. Gene names were assigned according to Ensembl Genes V75. Downstream analyses were performed in R v3.3.2 and with the Bioconductor package edgeR v3.14.0([9](#_ENREF_9)). Reads were normalized for sequencing depth and corrected for inter-sample dispersion using the edgeR functions “calcNormFactors” and “estimateTagwiseDisp”, resulting in counts-per-million (CPM) per gene. Differential gene expression analysis was performed with the edgeR functions “glmQLFit” and “glmQLFTest”, which reports p-value, false-discovery rate (FDR) and log-fold changes for each pair-wise comparison of B-ALL vs. T-ALL, T-ALL vs. ETP-ALL and B-ALL vs. ETP-ALL.

PCA (Principal Component Analysis) plots were generated of the CPM values using the prcomp function in R (center=TRUE; scale=TRUE) for all expressed genes (CPM > 1) and in a selection of genes from known target genes of the Notch and IL-7 signaling pathways (Sup. Fig.1).

For the microarray expression analysis in DBZ treated mice data was taken from dataset GSE71087. Data was processed using R Bioconductor package affy (v1.52.0), and annotated probes using R annotation database “mouse4302.db”. Data was normalized using RMA normalization, and differential expression was performed using lmFit and eBayes methods within affy package, followed by multiple testing corrections.

***In vivo* microscopy and Histology**

For intravital microscopy, images of the mouse calvarium were collected with Olympus FV1000 confocal/multiphoton microscope (ICBM Imaging Facility at IUSM) using a XLUMPLFL 20xW, NA 0.95 objective (Center Valley, PA). Hematopoietic cells were expressing GFP and vascular structures were delineated by dextran-Texas Red injected intravenously (20mg/ml, 100 µl) prior to imaging. Series of images through the depth of tissue (60μm Z-stacks at 1 μm interval and 512x512 pixel frame size) were collected from six regions of calvarium BM. Maximum Intensity Projection images were created with MetaMorph (Molecular Devices).

For histology, tissues (spleen, liver, and bone) were fixed with 4% paraformaldehyde (PFA/PBS), paraffin embedded, sectioned, and stained with hematoxylin-eosin. Bones were decalcified in 0.5mM EDTA prior to paraffin embedding.

**Study design**

The numbers of mice, cell donors, or cell line replicates were determined based on resource availability and previous or similar published work. In all experiments, animals and samples were randomly assigned to treatment groups. In animal studies the investigator was not blinded. Exact group, sample sizes and number of replicates for each experiment are indicated in each figure legend.

**Statistical analysis**

Descriptive statistics: number [n], mean, standard deviation, standard error, median (range) were used to summarize continuous variables/data; counts and percentages were used for categorical variables. All data meet the normality test (by Saphiro-wilk) and the equal variance test. Student t-test and ANOVA were used for mean comparisons between groups, using Tukey as post-hoc method for multiple comparison adjustment. Survival estimates were calculated based on the Kaplan-Meier product-limit method. 95% confidence intervals were calculated using the logit transformation and the Greenwood variance estimate. Differences between Kaplan-Meier curves were assessed by the log-rank test.

All calculations were performed using SAS® version 9.4 (SAS Institute, Cary, NC) or GraphPad Prism version 7. Statistical significance was set at the *P* <0.05 level.

Table S1. List of Antibodies

| **Flow Cytometry** | | |
| --- | --- | --- |
| **Ab** | **Vendor** | **Catalog #** |
| anti-CD3–FITC(17A2) | BD Biosciences | 555274 |
| anti-CD3–PE(145-2C11) | BD Biosciences | 553064 |
| anti-CD3-APC(145-2C11) | ebiociences | 17-0031-83 |
| anti-CD4-FITC (RM4-5) | BD Biosciences | 553047 |
| anti-CD4–PE (H129.19) | BD Biosciences | 553653 |
| anti-CD4–APC (G.K1.5) | ebiosciences | 17-0041-83 |
| anti-CD8–FITC (53-6.7) | BD Biosciences | 553031 |
| anti-CD8-PE(53-6.7) | BD Biosciences | 553032 |
| anti-CD8–eFluor780 (53-6.7) | ebiosciences | 47-0081-80 |
| anti-B220–PE (BA3-6B2) | BD Biosciences | 553090 |
| anti-Gr1–PE (RB6-8C5) | BD Biosciences | 553128 |
| anti-Mac1-PE(M1/70) | BD Biosciences | 553311 |
| anti-Mac1–APC (M1/70) | BD Biosciences | 553312 |
| anti-Ter119-PE | BD Biosciences | 553673 |
| anti-Sca1-APC (D7) | ebiosciences | 17-5981-83 |
| anti-c-Kit-eFluor780 (2B8) | Invitrogen | 47-1171-82 |
| anti-IL7Rα-PECy7 (A7R34) | ebiosciences | 25-1271-82 |
| anti- CD45.2-PECy5.5 (104) | ebiosciences | 45-0454-82 |
| anti-CD44-PE (IM7) | BD Biosciences | 553134 |
| anti-CD25-PECy5.5 (PC61.5) | ebiosciences | 45-0251-80 |
| anti-NK1.1-APC (PK136) | BD biosciences | 550627 |
| anti-TCRγδ-PE (GL3) | BD Biosciences | 553178 |

| **Immuno Blot and ChIP assay** | | |
| --- | --- | --- |
| **Ab** | **Vendor** | **Catalog #** |
| anti-β-Actin (I-19) | Santa Cruz Biotechnology | sc-1616 |
| anti-p27 (C19) | Santa Cruz Biotechnology | sc-528 |
| anti-p27 (D37H1) | Cell Signaling | 3688S |
| anti-SKP2 (H-435) | Santa Cruz Biotechnology | sc-7164 |
| anti-SKP2 (L70) | Cell Signaling | 41313S |
| anti-Notch1 (C-20-R) | Santa Cruz Biotechnology | sc-6014-R |
| anti-Jagged1 (H-114) | Santa Cruz Biotechnology | sc-8303 |
| **Immunohistochemistry** | | |
| **Ab** | **Vendor** | **Catalog #** |
| anti-p27 (D69C12) | Cell signaling | 3686S |
| anti-hCD45-FITC (2D1) | Invitrogen | 11-9459-42 |
| Anti-rabbit-AF555 | Invitrogen | A21428 |

Table S2. Primers for quantitative RT-PCR

| GAPDH mouse-F | AAGCCCATCACCATCTTCCA |
| --- | --- |
| GAPDH mouse-R | TAGACTCCACGACATACTCA |
| SKP2 mouse-F | TGGGATCTTTTCCTGTCTGTG |
| SKP2 mouse -R | TACCCGGAAAGAGCTGAAGC |
| Deltex mouse -F | GCCATGTACTCCAATGGCAACAAG |
| Deltex mouse -R | CGGGATGAGGTGAAACTCCATCTT |
| Hey1 mouse -F | tgagctgagaaggctggtac |
| Hey1 mouse -R | accccaaactccgatagtcc |
| SKP2 human-F | AACCTCTCCTGGTGTTTTGATTTC |
| SKP2 human-R | GTGGGAATTTCTCCAAGTTCAAGT |
| Notch1 human-F | GCCTTGCTGCCAGCGCCC |
| Notch1 human-R | CCAGTGGCTGCACGTCTGC |
| GAPDH human-F | AATCCCATCACCATCTTCCA |
| GAPDH human-R | TGGACTCCACGACGTACTCA |
| SKP2 human promoter-F | CTAGCAACGTTCCATCACCA |
| SKP2 human promoter-R | GGGAAAAGGAAAACCTGCTC |

**Supplementary Figure 1. Loss of SKP2 does not affect thymocytes differentiation.**

(A) Fold change for *Skp2* mRNA levels measured by q-RT-PCR in BM, Thymus, Spleen, Liver, PB, Lung and Kidney (left panel; each group n=3). Right panel: *Skp2* gene expression was plotted from the publically available Immunological Genome Project data (Immgen.com), obtained using mouse sorted thymic cell subsets probed with Affymetrix Mouse Gene 1.0ST arrays.

(B) Fold change for *Skp2* mRNA levels measured in BM cells by q-RT-PCR in Skp2^+/+^ (black) Skp2^+/-^ (purple) and Skp2^-/-^ (red) mice (left panel; each group n=6). Right panel shows SKP2 protein levels in the BM in a representative experiment.

(C) Absolute number of thymocytes from *Skp2*^+/+^ and *Skp2*^-/-^ mice; each group n=4.

(D) Flow cytometry gating strategy for analysis of thymocytes populations. Left panel: scheme of the gating strategy. Right panel: Plots representative for 3 *Skp2^+/+^* (# 1, 2 and 3) and 3 *Skp2^-/-^* mice (#4, 5 and 6).

(E) *Skp2* gene expression was plotted from the publically available Immunological Genome Project data (Immgen.com), obtained using mouse peripheral T-cells subsets probed with Affymetrix Mouse Gene 1.0ST arrays.

(F) Absolute number of Sp cells from *Skp2*^+/+^ and *Skp2*^-/-^ mice expressing CD3 (left panel), γɖ cells (right panel), and NK1.1 (far right panel); each group n=3.

In bar graphs, results are shown as average±SD. In scatter graph, average is shown with horizontal line. Paired T-test or one-way ANOVA was used for statistics. ***p<0.005.

**Supplementary Figure 2. Effect of Notch inhibition on *Skp2* expression in vivo.**

(A) Heat map representing color-coded expression levels of Notch, Hes1 and SKP2 in Notch-driven T-ALL leukemic cells from mice treated with DMSO (black) or DBZ (purple) in vivo (n=3).

(D) Percentage of cells in S-phase measured by BrdU incorporation in *Rbpj^+/+^* or *Rbpj^-/-^* total thymocytes, fresh (Day0), and co-cultured with OP9-Dll1 cells in the presence of IL-7 (25ng/ml) for 4 days (Day4). Results are indicated as average±SE; n=4 for (*Rbpj^-/-^) and* n=6 (*Rbpj^+/+^*) in 2 independent experiments.

*p<0.05, **p<0.01, ***p<0.005 by t-test or one-way ANOVA.

**Supplementary Figure 3. Notch-induced T-ALL model.**

Lin^-^ cells from C57Bl/6 mice (CD45.2) were transduced with MSCV-GFP (Vector) or MSCV-ICN1-GFP (ICN) constructs. 2.5x10^4^ ICN/GFP^+^ cells were transplanted into lethally irradiated BoyJ (CD45.1). Recipients were analyzed for:

(A) Intravital two-photon imaging of calvarium BM of healthy mice (transplanted with GFP^+^ cells) and leukemic mice (transplanted with ICN-GFP+ cells) at week 8; donor Vector GFP^+^ and ICN GFP^+^ cells are in green, vascular structures in red. Representative image, 20x magnification.

(B) Frequency of GFP^+^ cells (left) and DP T-cells (CD4^+^CD8^+^; right) in gated CD45.2^+^ donor cells in the BM; n=4 (vector) and n=5 (ICN).

(C) Frequency of DP T-cells (CD4^+^CD8^+^) in gated CD45.2^+^ donor cells at week 8 in the PB; n=6 (vector) and n=10 (ICN).

(D) Scatter graph (left panel) of spleen weight (n=5 vector and n=6 ICN) and representative picture of spleens in mice transplanted with Vector or ICN donor cells (right panel).

(E) Fold change for human *Notch1-ICN* mRNA expression measured by qRT-PCR in the BM (left panel; n=4) and spleen (right panel; n=6 (vector) and n=10 (ICN)).

(F) CHIP assay. DNA isolated from the thymus of mice transplanted with ICN GFP^+^ cells was immunoprecipitated with anti-Notch1 (N1) or anti-Jagged (irrelevant) antibodies. Images shows detection of *Skp2* promoter region containing the Notch/RBPJ binding sequence sites by PCR, representative experiment.

All results are from 2 independent experiments. In scatter plots average is shown by a horizontal line. *p<0.05, ***p<0.005 by t-test.

**Supplementary Figure 4. Effects of SKP2 loss on Notch-induced leukemia.**

Lin^-^ cells from *Skp2*^+/+^, *Skp2*^+/-^, or *Skp2*^-/-^ (CD45.2) mice were transduced with MSCV-GFP (Vector), MSCV-ICN1-GFP (ICN) or MSCV-N1ΔEGFΔLNRΔP-GFP (N1ΔEGF) constructs. 2.5x10^4^ GFP^+^ cells were transplanted into lethally irradiated BoyJ (CD45.1) and recipients (in 3 independent experiments) were analyzed for:

(A) WBC count in PB. *Skp2*^+/+^ Vector (n=6), *Skp2*^+/+^ ICN (n=10), for *Skp2*^+/-^ ICN (n=10) and *Skp2*^-/-^ ICN (n=6).

(B) SKP2, p27^Kip1^ and β-Actin protein levels by Western blot in spleen extracts; thymus is used as control for SKP2 expression; *Skp2*^-/-^ samples were confirmed negative by RT-PCR.

(C) Dot blots (left panel) and bar graph (right panel) show BrdU incorporation and DNA content for early and late S-phase in GFP^+^ cells from BM *Skp2^+/+^* ICN (n=5) and *Skp2^-/-^* ICN (n=3)

(D) Survival probabilities.

All results are expressed as average±SE from 2 independent experiments. *p<0.05, ***p<0.005 by two-sample t-test or t-test.

**Supplementary Figure 5. Evolution of SKP2-null ICN cells on Notch-induced leukemia.**

Lin^-^ cells from *Skp2*^+/+^, *Skp2*^+/-^, or *Skp2*^-/-^ (CD45.2) mice were transduced with MSCV-GFP (Vector) or MSCV-ICN1-GFP (ICN) constructs. 2.5x10^4^ GFP^+^ cells were transplanted into lethally irradiated BoyJ (CD45.1).

(A) Bar graph represents the engraftment of recipient animals as percentage of CD45.2% in PB at the time points indicated. *Skp2*^+/+^ Vector (n=6), *Skp2*^+/+^ ICN (n=10) and*Skp2*^-/-^ ICN (n=6).

(B) Fold change for *Skp2* mRNA levels measured by q-RT-PCR in BM cells. Analysis was performed at time of death in mice that developed leukemia and at one year from transplant in mice that did not. *Skp2*^+/+^ Vector (n=3), *Skp2*^+/+^ ICN (n=5), *Skp2*^+/-^ ICN (n=3) and *Skp2*^-/-^ ICN n=4).

(C) Fold change for *human Notch1-ICN* mRNA levels measured by qRT-PCR in transduced Lin^-^ cells prior to transplant. *Skp2*^+/+^ Vector (n=3), *Skp2*^+/+^ ICN (n=4), *Skp2*^+/-^ ICN (n=3) and *Skp2*^-/-^ ICN (n=4).

(D) Absolute number of GFP^+^ gated on CD45.2^+^ cells per femur engrafted in the BM of recipient mice at 2.5 weeks; n=2.

(E) Fold change for *human Notch1-ICN* mRNA levels measured by q-RT-PCR in BM cells. Analysis was performed at time of death in mice that developed leukemia and at one year from transplant in mice that did not. Samples in which Notch1 was not detected are shown as 0. *Skp2*^+/+^ Vector (n=3) *Skp2*^+/+^ ICN (n=6), *Skp2*^-/+^ ICN (n=4), *Skp2^-^*^/-^ = (n=3) and *Skp2^-^*^/-^ ICN^s^ (n=3).

All experiments are shown as average±SD. In scatter graph, average is shown with horizontal line. *p<0.05 by one-way ANOVA.

**Supplementary Figure 6. Figure 5 continuation. Evolution of SKP2-null ICN cells in vivo.**

(A) Bar graphs summarize average percentage of the indicated BM populations in gated CD45.2^+^ cells. *Skp2*^+/+^ Vector (n=6), *Skp2*^+/+^ ICN (n=10), *Skp2*^+/-^ ICN (n=4), *Skp2^-/-^* ICN^ns^ (n=4), and *Skp2*^-/-^ ICN^s^ (n=4).

(B) Scatter graph shows average Spleen weight. n=6 for *Skp2*^+/+^ vector, *Skp2*^+/+^ ICN and *Skp2^-/-^* ICN^ns^, n=4 for *Skp2*^+/-^ ICN and n=5 for *Skp2*^-/-^ ICN^s^.

(C) Histological analysis of parenchymal organs from spleen, liver, and bone (representative samples at 10X) performed at time of death in mice that developed leukemia and at one year from transplant in mice that did not.

(D) Kaplan-Meier survival curve in secondary transplants. Recipients received 5x10^5^ BM cells from leukemic primary recipients (*Skp2^+/+^* ICN or *Skp2^-/-^* ICN; each group n=5).

All experiments are shown as average±SD. In scatter graph, average is shown with horizontal line *p<0.05, **p<0.01, ***p<0.005 by one-way ANOVA. p0.035 by log-rank test in survival curve.

**Supplementary Figure 7. Notch/IL-7 target genes.**

(A) Heat map representing color-coded expression levels of 23 known downstream Notch and IL-7 signaling pathways that are differentially expressed in T-ALL (dark blue), ETP-ALL (blue) and B-ALL (purple). These genes were used to perform PCA analysis (Fig.6C).

**Supplementary Figure 8. SKP2 blockade by C1 in primary murine BM cells.**

Viability assays for the C1 inhibitor were performed at 96h for cell lines and 48h for primary cells. DMSO was used as vehicle control in all experiments.

(A) Percentage viability of *Skp2*^+/+^ (black) or *Skp2*^-/-^ (red) MEFs treated with increasing doses of the SKP2 inhibitor C1 (each group n=4 in 2 independent experiments).

(B) Percentage viability of murine primary leukemia cells (derived from ICN recipients) treated with increasing doses of the SKP2 inhibitor C1 (n=3).

(C) Percentage viability in murine primary healthy BM cells (n=2) and in primary murine leukemia cells (derived from ICN recipients; n=3) treated with 0.625 and 1.25µM of the SKP2 inhibitor C1.

(D) Colony forming units from murine primary healthy BM cells treated with 1.25µM C1 or DMSO (n=2).

(E) SKP2, p27^Kip1^, and β-Actin protein levels by Western blot in T-cell lines; representative of 2 experiments.

(F) SKP2, p27^Kip1^, and β-Actin protein levels by Western blot in TAIL7 cell lines at 6, 24 and 48h following IL-7 stimulation (10ng/ml); representative of 3 experiments.

All experiments are shown as average±SD. *p<0.05, by t-test.

**Supplementary Figure 9. Effects of SKP2 inhibition by C25 in a xenograft model of T-ALL**

(A) Percentage viability of TAIL7 cells treated with increasing doses of the SKP2 inhibitor C25 for 96 hours (average±SE); n=3 in 2 independent experiments.

(B) Scheme summarizes xenograft model and therapeutic regimen. 3x10^6^ TAIL7-ICN/GFP^+^ cells were transplanted into NSG mice. Treatment with 50mg/kg of C25 or vehicle was administered by oral gavage (OG) 3 times/week for 4 weeks and was started when blasts in the PB reached around 2-5%, 4 weeks after transplant. 1 animal in the study started with IP treatment and was switched to oral gavage soon after (IP-OG); n=4 mice/group.

(C) Representative plots show ICN-GFP^+^ blasts before treatment at week 4 from transplant and during treatment at week 7.

(D) Average percentage of ICN-GFP^+^ blasts in the PB at week 7 (n=4).

(E) Average percentage of ICN-GFP^+^ blasts in the spleen at end point (n=4).

Results are shown as average±SD. *p<0.05, ***p<0.005 by t-test.


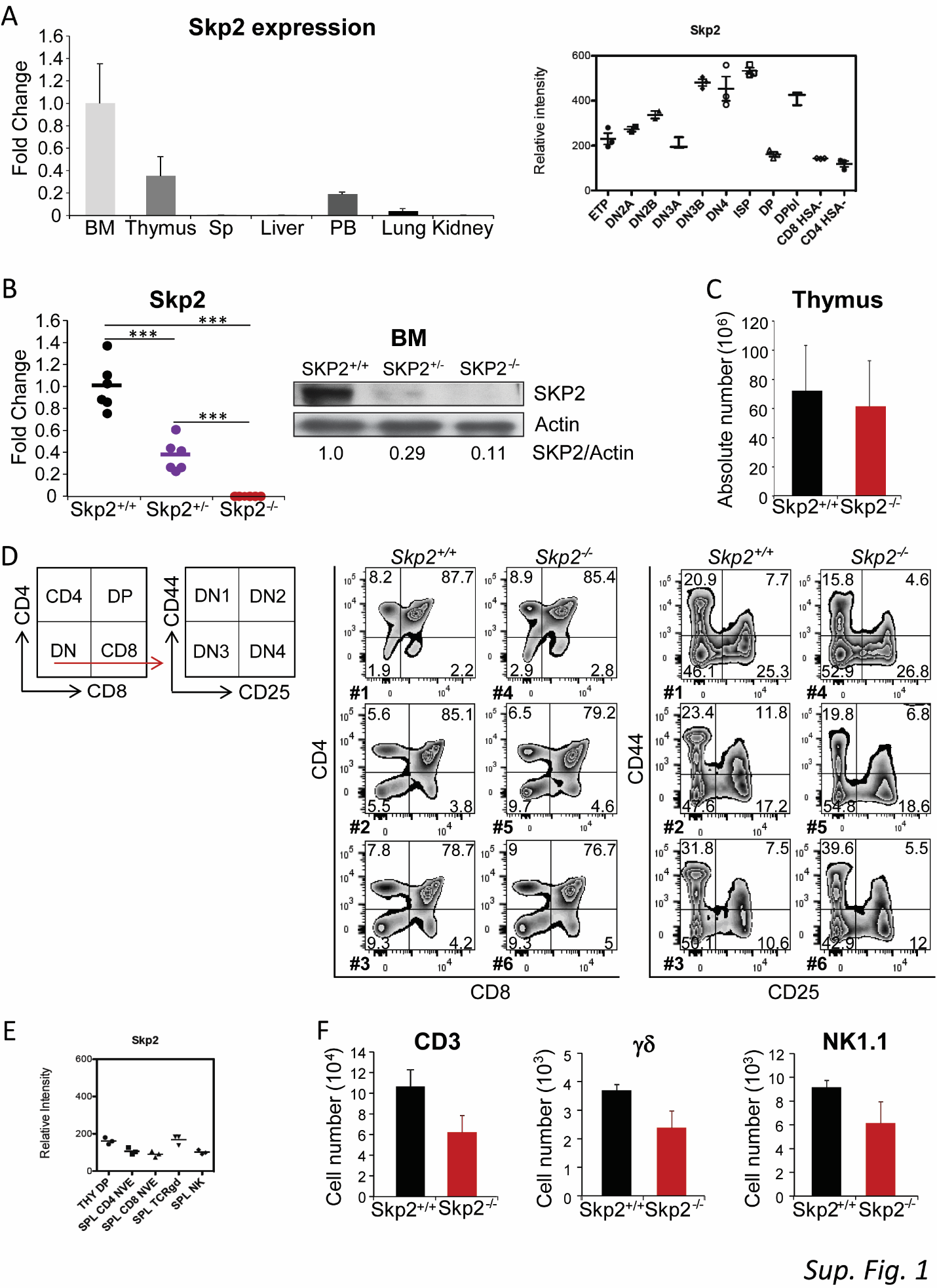

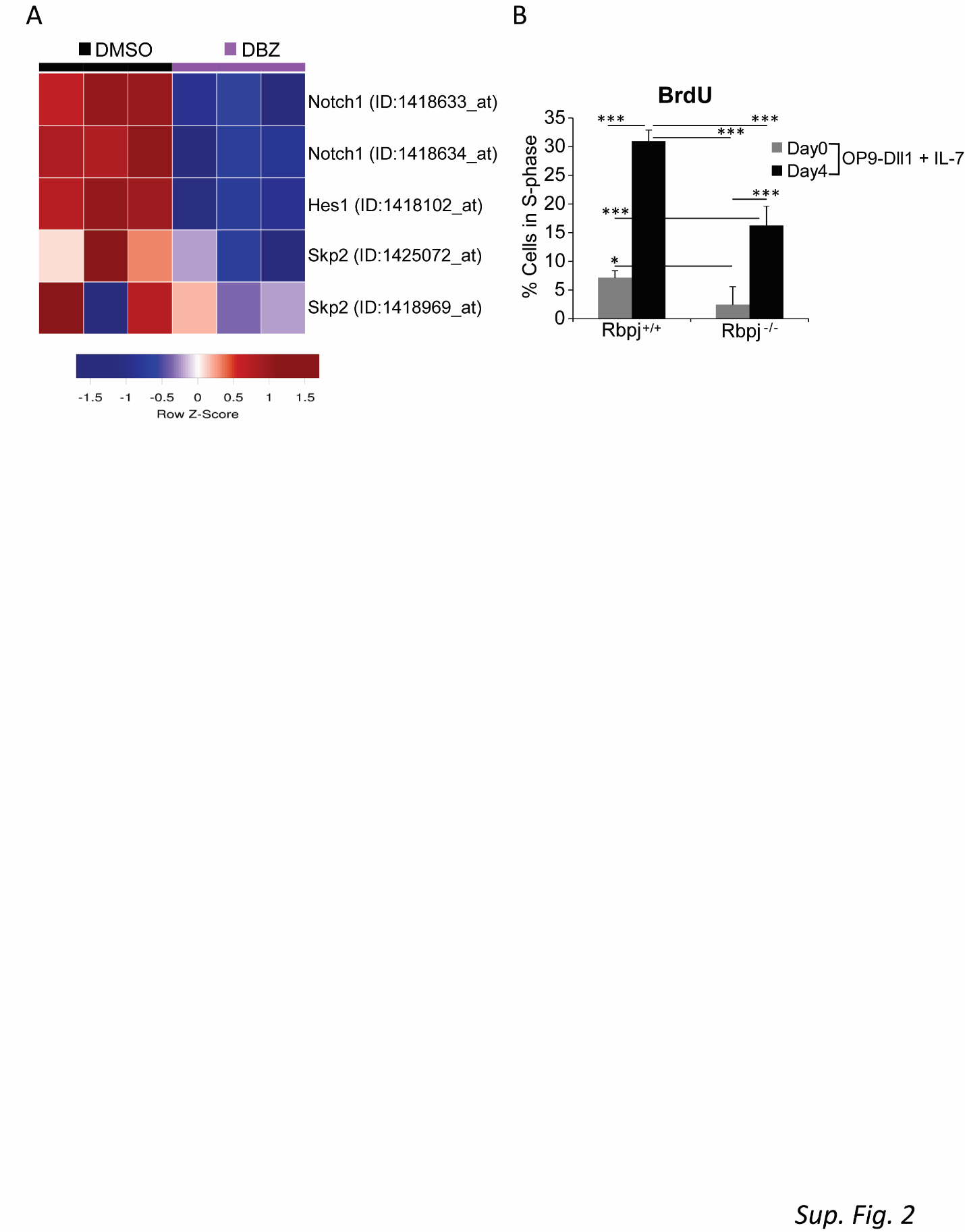

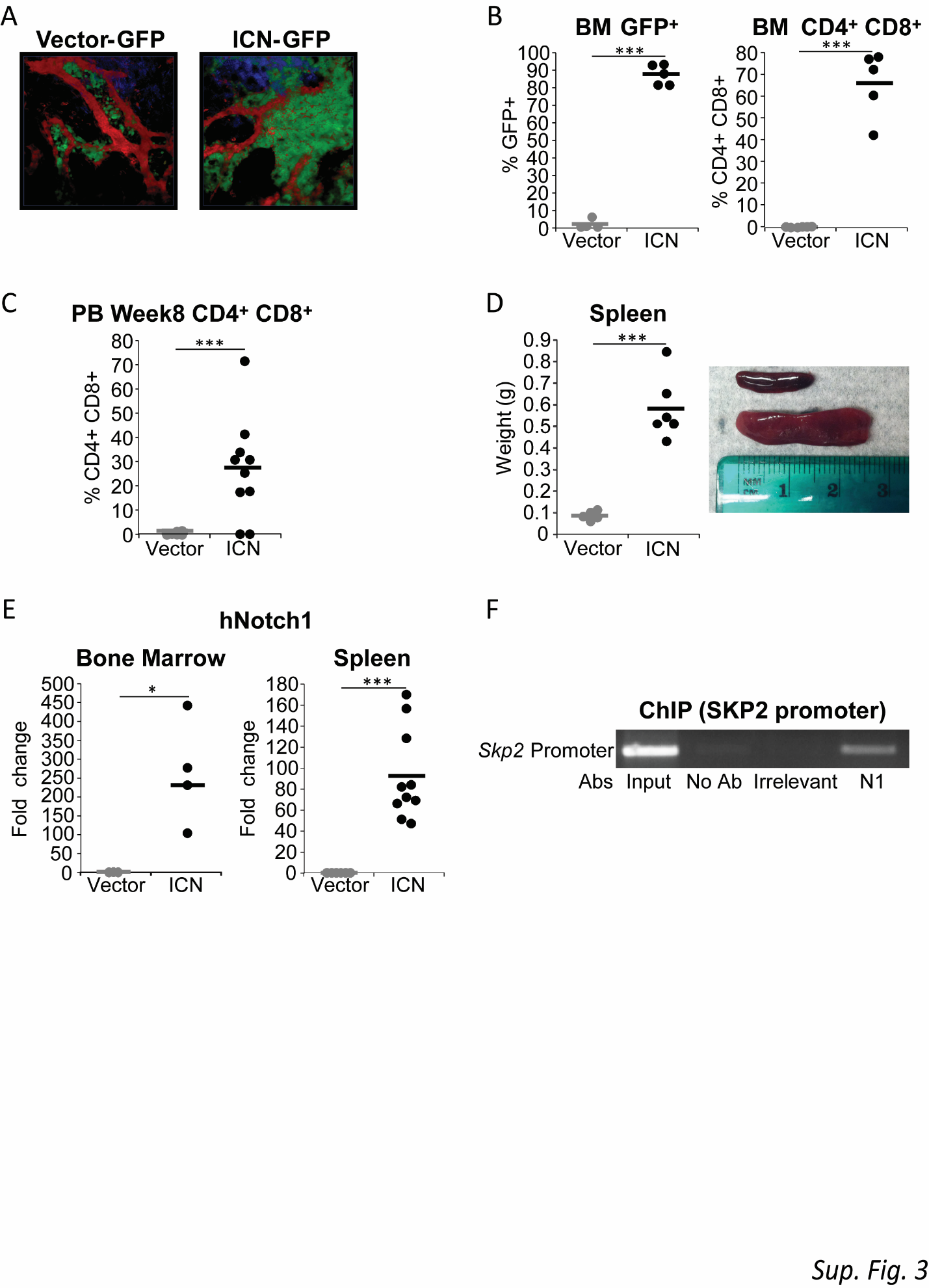

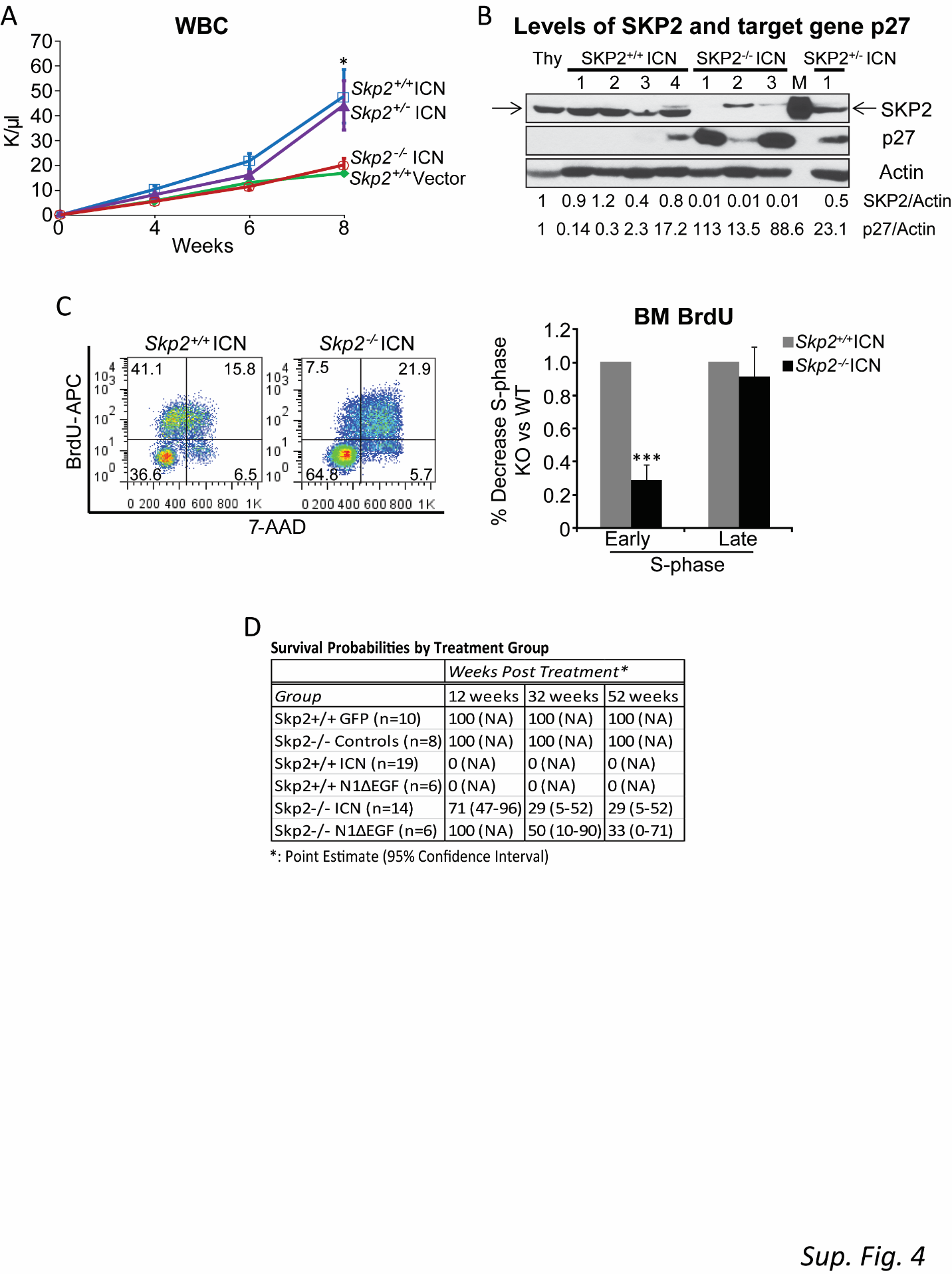

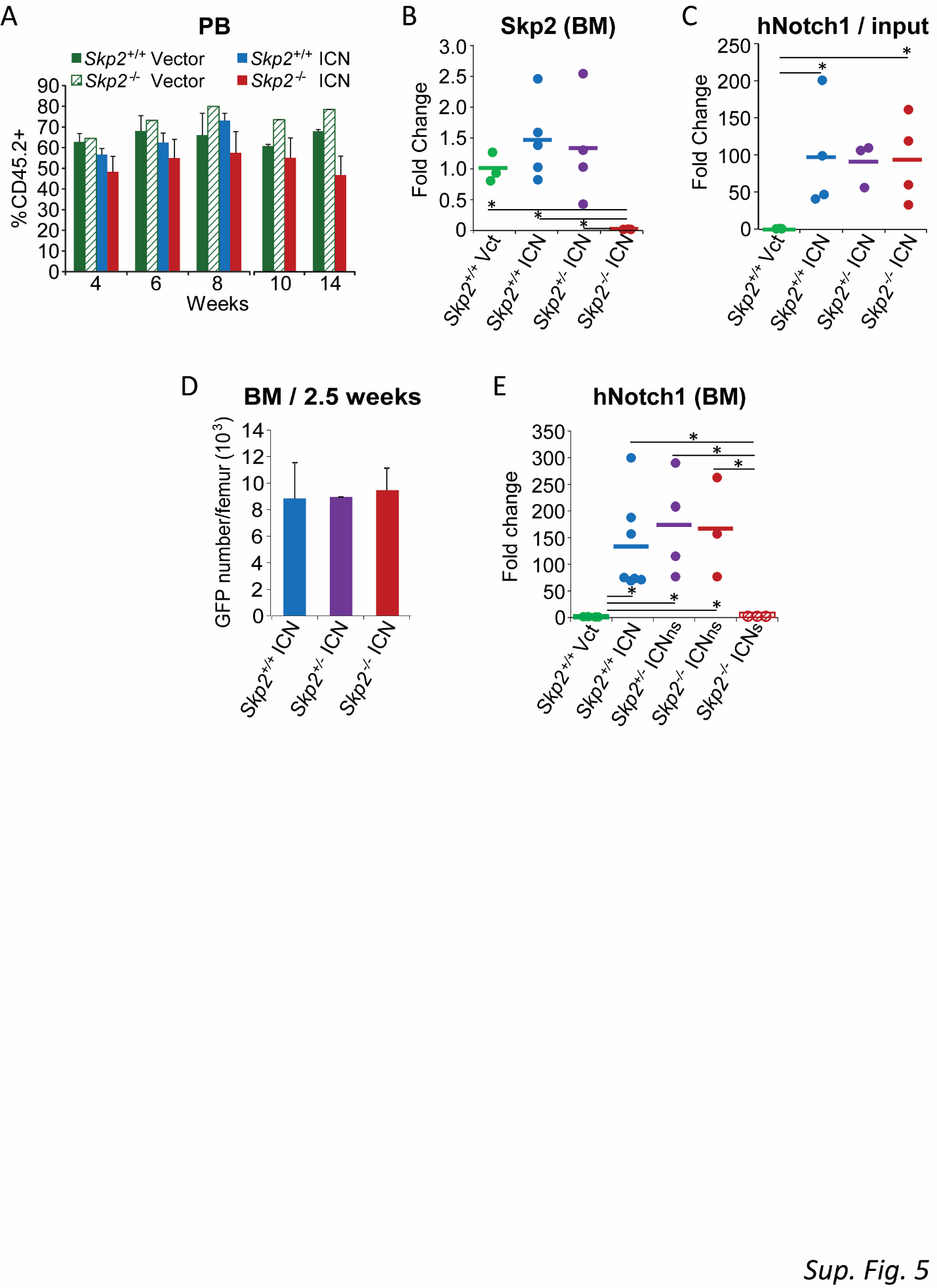

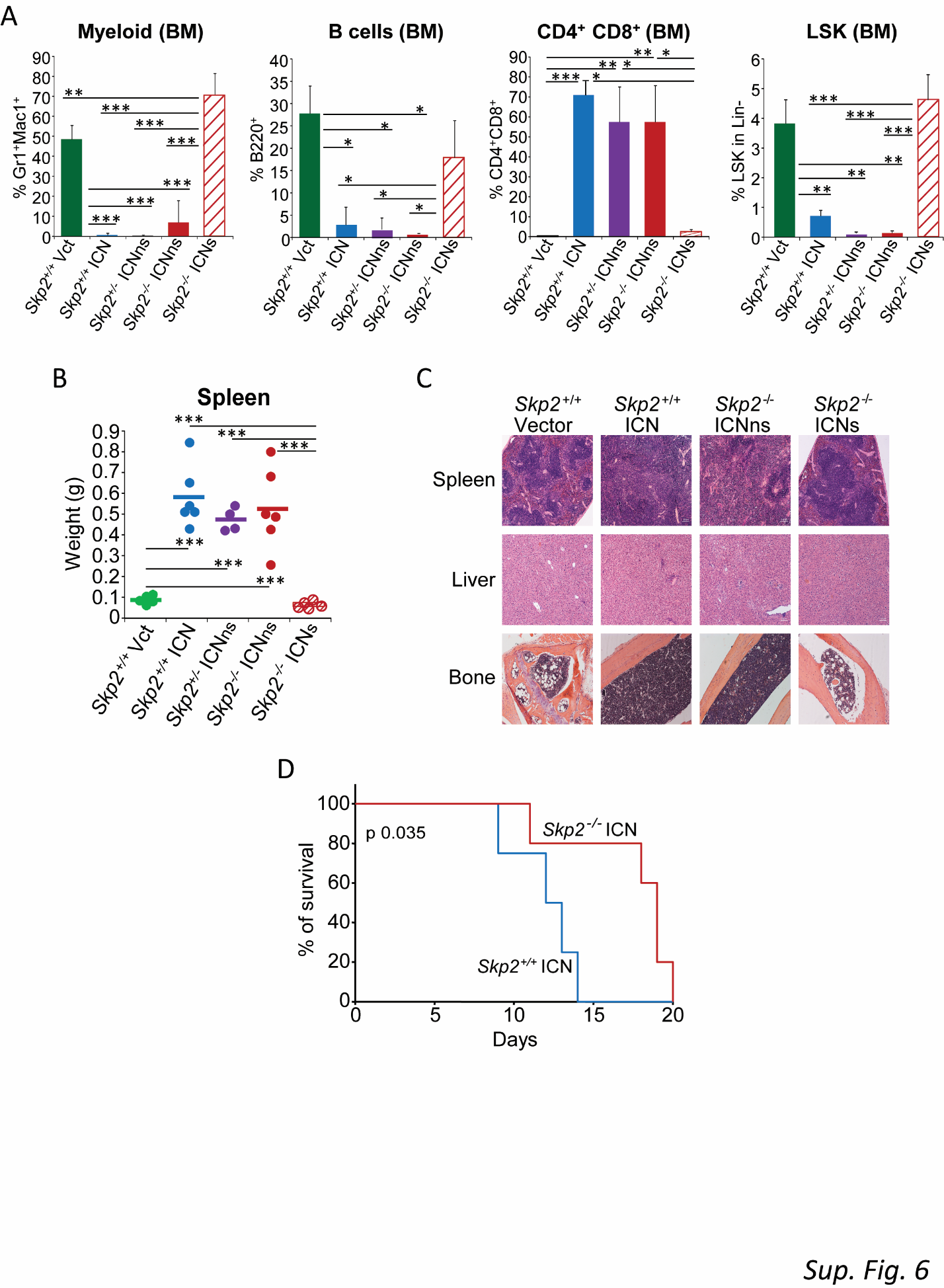

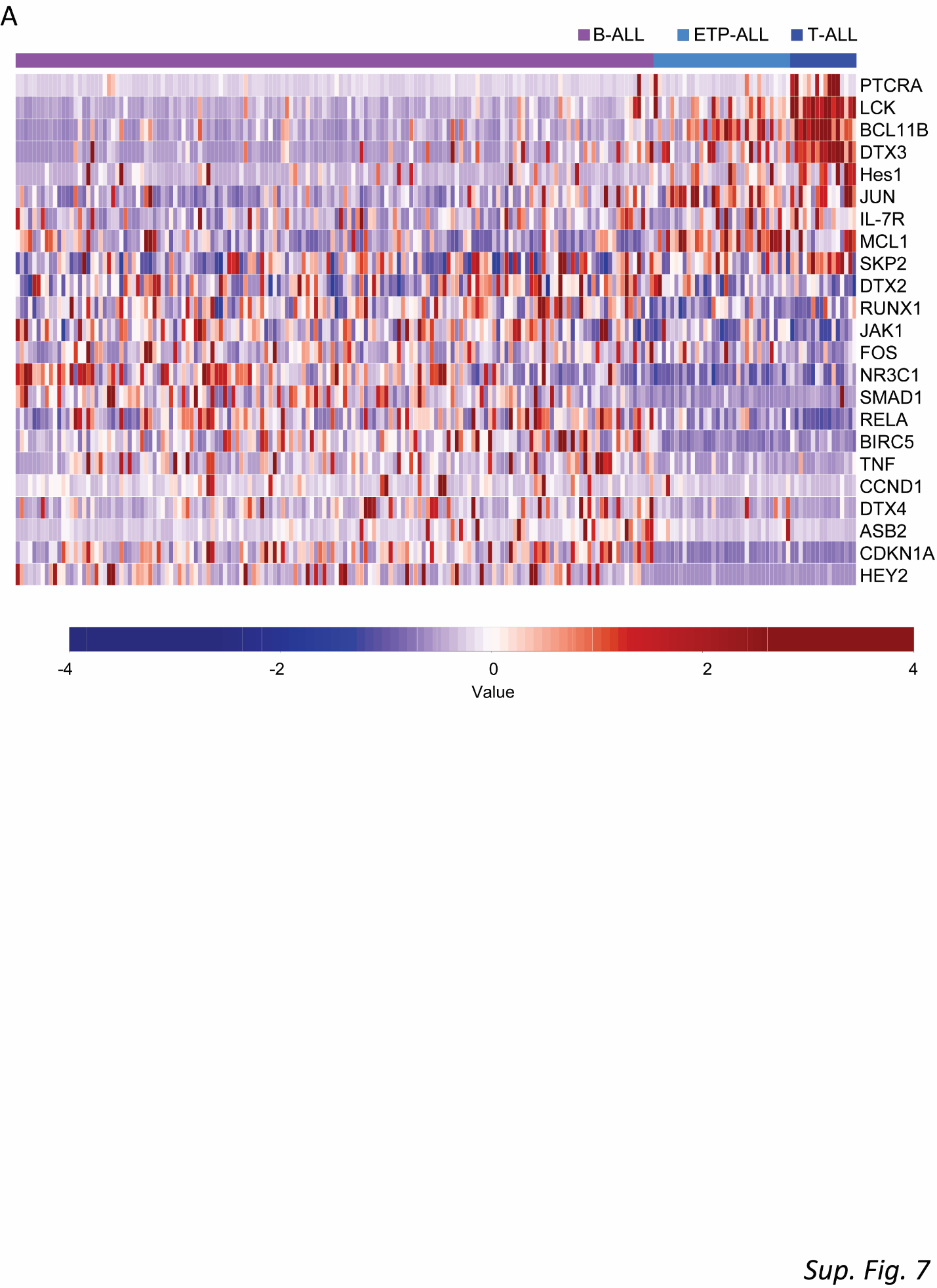

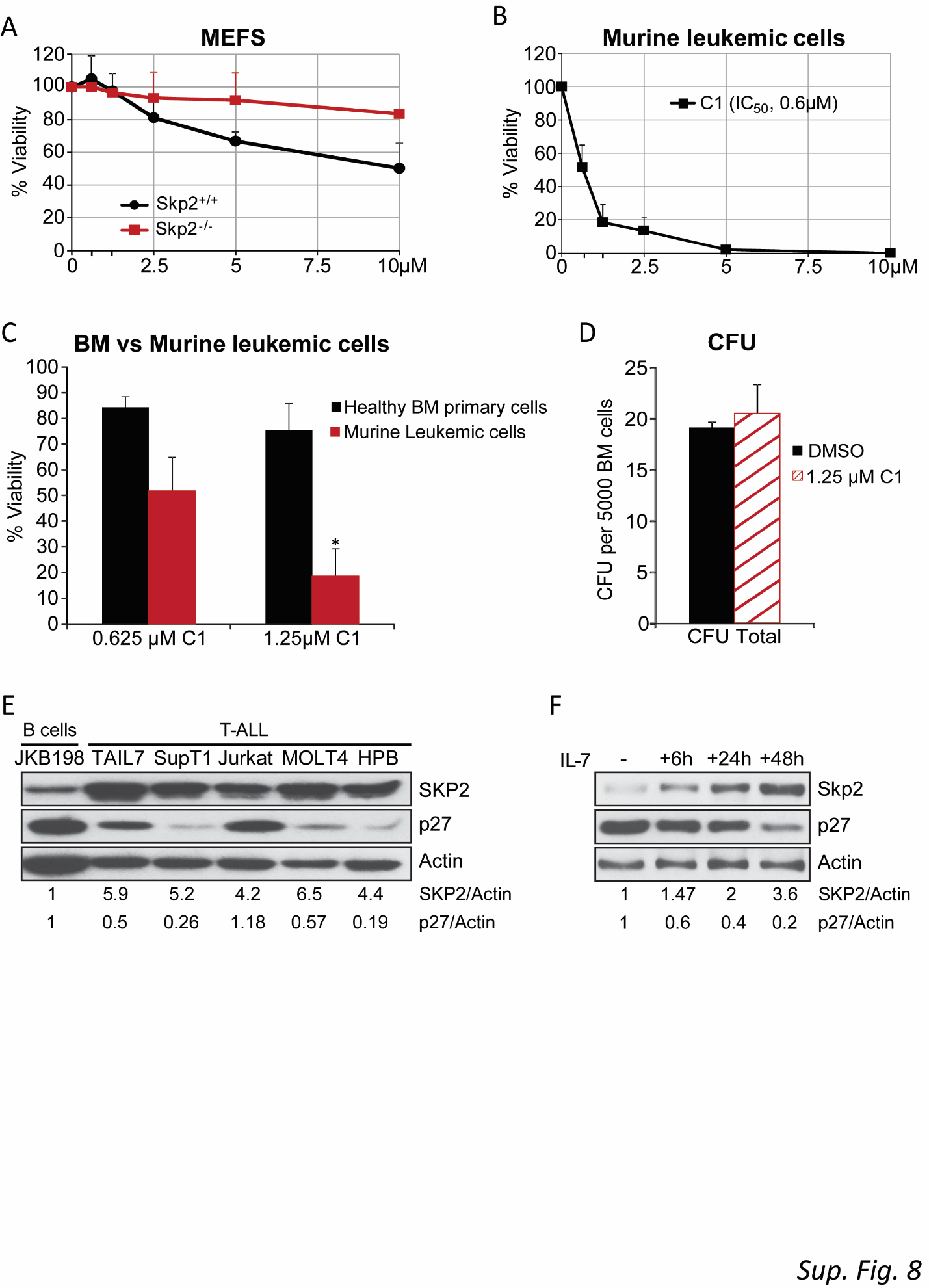

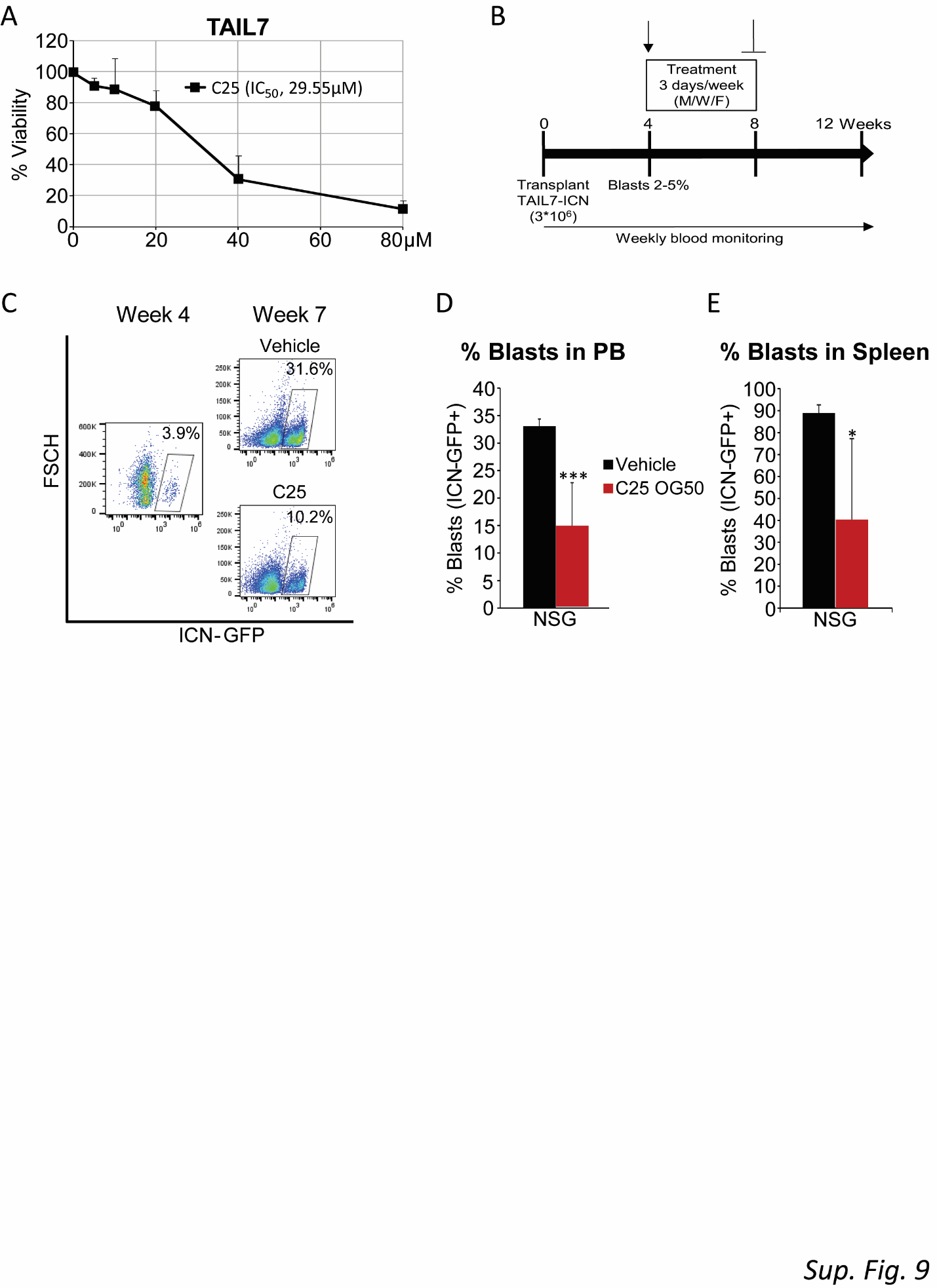


1. Abbondanzo SJ, Gadi I, Stewart CL. Derivation of embryonic stem cell lines. Methods in enzymology. 1993;225:803-23. PubMed PMID: 8231888.

2. Barata JT, Boussiotis VA, Yunes JA, Ferrando AA, Moreau LA, Veiga JP, et al. IL-7-dependent human leukemia T-cell line as a valuable tool for drug discovery in T-ALL. Blood. 2004 Mar 1;103(5):1891-900. PubMed PMID: 14615384.

3. Yashiro-Ohtani Y, He Y, Ohtani T, Jones ME, Shestova O, Xu L, et al. Pre-TCR signaling inactivates Notch1 transcription by antagonizing E2A. Genes Dev. 2009 Jul 15;23(14):1665-76. PubMed PMID: 19605688. Pubmed Central PMCID: 2714710. Epub 2009/07/17. eng.

4. Rodriguez S, Wang L, Mumaw C, Srour EF, Lo Celso C, Nakayama K, et al. The SKP2 E3 ligase regulates basal homeostasis and stress-induced regeneration of HSCs. Blood. 2011 Jun 16;117(24):6509-19. PubMed PMID: 21502543. Pubmed Central PMCID: 3123021.

5. Mehrotra P, Riley JP, Patel R, Li F, Voss L, Goenka S. PARP-14 functions as a transcriptional switch for Stat6-dependent gene activation. J Biol Chem. 2011 Jan 21;286(3):1767-76. PubMed PMID: 21081493. Pubmed Central PMCID: 3023471.

6. Heng TS, Painter MW, Immunological Genome Project C. The Immunological Genome Project: networks of gene expression in immune cells. Nat Immunol. 2008 Oct;9(10):1091-4. PubMed PMID: 18800157.

7. Coustan-Smith E, Mullighan CG, Onciu M, Behm FG, Raimondi SC, Pei D, et al. Early T-cell precursor leukaemia: a subtype of very high-risk acute lymphoblastic leukaemia. The Lancet Oncology. 2009 Feb;10(2):147-56. PubMed PMID: 19147408. Pubmed Central PMCID: 2840241.

8. Dobin A, Davis CA, Schlesinger F, Drenkow J, Zaleski C, Jha S, et al. STAR: ultrafast universal RNA-seq aligner. Bioinformatics. 2013 Jan 1;29(1):15-21. PubMed PMID: 23104886. Pubmed Central PMCID: 3530905.

9. Robinson MD, McCarthy DJ, Smyth GK. edgeR: a Bioconductor package for differential expression analysis of digital gene expression data. Bioinformatics. 2010 Jan 1;26(1):139-40. PubMed PMID: 19910308. Pubmed Central PMCID: 2796818.
